# Reversed oxidative TCA (roTCA) for carbon fixation by an *Acidimicrobiia* strain from a saline lake

**DOI:** 10.1093/ismejo/wrae147

**Published:** 2024-07-29

**Authors:** Lei Gao, Lan Liu, Ai-Ping Lv, Lin Fu, Zheng-Han Lian, Takuro Nunoura, Brian P Hedlund, Qing-Yu Xu, Dildar Wu, Jian Yang, Mukhtiar Ali, Meng-Meng Li, Yong-Hong Liu, André Antunes, Hong-Chen Jiang, Lei Cheng, Jian-Yu Jiao, Wen-Jun Li, Bao-Zhu Fang

**Affiliations:** Key Laboratory of Ecological Safety and Sustainable Development in Arid Lands, Xinjiang Institute of Ecology and Geography, Chinese Academy of Sciences, Urumqi, 830011, PR China; State Key Laboratory of Biocontrol, Guangdong Provincial Key Laboratory of Plant Stress Biology and Southern Marine Science and Engineering Guangdong Laboratory (Zhuhai), School of Life Sciences, Sun Yat-Sen University, Guangzhou, 510275, PR China; State Key Laboratory of Biocontrol, Guangdong Provincial Key Laboratory of Plant Resources and Southern Marine Science and Engineering Guangdong Laboratory (Zhuhai), School of Life Sciences, Sun Yat-Sen University, Guangzhou 510275, PR China; State Key Laboratory of Biocontrol, Guangdong Provincial Key Laboratory of Plant Resources and Southern Marine Science and Engineering Guangdong Laboratory (Zhuhai), School of Life Sciences, Sun Yat-Sen University, Guangzhou 510275, PR China; Key Laboratory of Development and Application of Rural Renewable Energy, Biogas Institute of Ministry of Agriculture and Rural Affairs, Chengdu 610000, PR China; State Key Laboratory of Biocontrol, Guangdong Provincial Key Laboratory of Plant Resources and Southern Marine Science and Engineering Guangdong Laboratory (Zhuhai), School of Life Sciences, Sun Yat-Sen University, Guangzhou 510275, PR China; Research Center for Bioscience and Nanoscience (CeBN), Japan Agency for Marine-Earth Science and Technology, Yokosuka 237-0061, Japan; School of Life Sciences, University of Nevada Las Vegas, Las Vegas, NV 89154, United States; Nevada Institute of Personalized Medicine, University of Nevada Las Vegas, Las Vegas, NV 89154, United States; Key Laboratory of Ecological Safety and Sustainable Development in Arid Lands, Xinjiang Institute of Ecology and Geography, Chinese Academy of Sciences, Urumqi, 830011, PR China; State Key Laboratory of Biocontrol, Guangdong Provincial Key Laboratory of Plant Stress Biology and Southern Marine Science and Engineering Guangdong Laboratory (Zhuhai), School of Life Sciences, Sun Yat-Sen University, Guangzhou, 510275, PR China; State Key Laboratory of Biocontrol, Guangdong Provincial Key Laboratory of Plant Resources and Southern Marine Science and Engineering Guangdong Laboratory (Zhuhai), School of Life Sciences, Sun Yat-Sen University, Guangzhou 510275, PR China; Key Laboratory of Biogeology and Environmental Geology, China University of Geosciences, Wuhan 430074, PR China; Advanced Water Technology Laboratory, National University of Singapore (Suzhou) Research Institute, Suzhou, Jiangsu 215123, PR China; State Key Laboratory of Biocontrol, Guangdong Provincial Key Laboratory of Plant Resources and Southern Marine Science and Engineering Guangdong Laboratory (Zhuhai), School of Life Sciences, Sun Yat-Sen University, Guangzhou 510275, PR China; Key Laboratory of Ecological Safety and Sustainable Development in Arid Lands, Xinjiang Institute of Ecology and Geography, Chinese Academy of Sciences, Urumqi, 830011, PR China; State Key Laboratory of Lunar and Planetary Sciences, Macau University of Science and Technology, Taipa, Macau SAR 999078, PR China; Key Laboratory of Ecological Safety and Sustainable Development in Arid Lands, Xinjiang Institute of Ecology and Geography, Chinese Academy of Sciences, Urumqi, 830011, PR China; Key Laboratory of Biogeology and Environmental Geology, China University of Geosciences, Wuhan 430074, PR China; Key Laboratory of Development and Application of Rural Renewable Energy, Biogas Institute of Ministry of Agriculture and Rural Affairs, Chengdu 610000, PR China; State Key Laboratory of Biocontrol, Guangdong Provincial Key Laboratory of Plant Resources and Southern Marine Science and Engineering Guangdong Laboratory (Zhuhai), School of Life Sciences, Sun Yat-Sen University, Guangzhou 510275, PR China; Key Laboratory of Ecological Safety and Sustainable Development in Arid Lands, Xinjiang Institute of Ecology and Geography, Chinese Academy of Sciences, Urumqi, 830011, PR China; State Key Laboratory of Biocontrol, Guangdong Provincial Key Laboratory of Plant Resources and Southern Marine Science and Engineering Guangdong Laboratory (Zhuhai), School of Life Sciences, Sun Yat-Sen University, Guangzhou 510275, PR China; Key Laboratory of Ecological Safety and Sustainable Development in Arid Lands, Xinjiang Institute of Ecology and Geography, Chinese Academy of Sciences, Urumqi, 830011, PR China

**Keywords:** Acidimicrobiia, carbon fixation, roTCA, CBB cycle, chemolithoautotrophic enrichment, metagenome, transcriptome, stable-isotope probing, ancestral character reconstruction

## Abstract

*Acidimicrobiia* are widely distributed in nature and suggested to be autotrophic via the Calvin–Benson–Bassham (CBB) cycle. However, direct evidence of chemolithoautotrophy in *Acidimicrobiia* is lacking. Here, we report a chemolithoautotrophic enrichment from a saline lake, and the subsequent isolation and characterization of a chemolithoautotroph, *Salinilacustristhrix flava* EGI L10123^T^, which belongs to a new *Acidimicrobiia* family. Although strain EGI L10123^T^ is autotrophic, neither its genome nor *Acidimicrobiia* metagenome-assembled genomes from the enrichment culture encode genes necessary for the CBB cycle. Instead, genomic, transcriptomic, enzymatic, and stable-isotope probing data hinted at the activity of the reversed oxidative TCA (roTCA) coupled with the oxidation of sulfide as the electron donor. Phylogenetic analysis and ancestral character reconstructions of *Acidimicrobiia* suggested that the essential CBB gene *rbcL* was acquired through multiple horizontal gene transfer events from diverse microbial taxa. In contrast, genes responsible for sulfide- or hydrogen-dependent roTCA carbon fixation were already present in the last common ancestor of extant *Acidimicrobiia*. These findings imply the possibility of roTCA carbon fixation in *Acidimicrobiia* and the ecological importance of *Acidimicrobiia*. Further research in the future is necessary to confirm whether these characteristics are truly widespread across the clade.

## Introduction

The class *Acidimicrobiia* is a deep-rooted lineage within the phylum *Actinomycetota* [1–6] that includes three validly published families under the International Code of Nomenclature of Prokaryotes (ICNP) (https://lpsn.dsmz.de/order/acidimicrobiales): *Acidimicrobiaceae*, *Iamiaceae*, and *Ilumatobacteraceae* [7, 8]. *Acidimicrobiaceae* are thermoacidophilic microbes capable of oxidizing/ reducing iron and sulfur compounds [3, 9, 10]. In contrast, *Iamiaceae* and *Ilumatobacteraceae* have mainly been found in marine environments and can grow under more moderate temperatures (15–55°C) as well as pH values (pH 6–11) [11, 12]. Despite the widespread occurrence of *Acidimicrobiia* in various habitats and some existing knowledge about them, the broad ecological characteristics of this taxon remain elusive, primarily due to the limited number and diversity of pure cultures as well as limited research focusing on their ecological functions [6, 13]. It has been reported that *Actinomycetota* contribute to the synthesis of organic matter through chemolithoautotrophy, with *Acidimicrobiia* playing a key role in this process [14–16]. Carbon fixation by chemolithoautotrophic microbes had a profound influence on the transition from the inorganic to the organic world via biological CO_2_ reduction, an indispensable process for sustaining life on Earth as it is accountable for the synthesis of the majority of organic carbon molecules [14, 17]. Probing the carbon fixation abilities of *Acidimicrobiia* is important for broadening our understanding of microbial diversity, carbon cycling, and biogeochemical processes. Bay et al. and Norris et al. reported several *Acidimicrobiia* metagenome-assembled genomes (MAGs) that encode genes for uptake hydrogenases and RuBisCO (including oxygen-producing photoautotrophic RuBisCO type IA and chemoautotrophic RuBisCO type IE), which suggested the potential for hydrogenotrophic carbon fixation by some *Acidimicrobiia* via the Calvin–Benson–Bassham (CBB) cycle [15, 16, 18, 19]. Although these studies provided valuable insights into their potential ability to fix carbon, no experimental evidence for carbon fixation in this class has been reported.

Here, we report a new isolate belonging to the class *Acidimicrobiia*, herein named *Salinilacustristhrix flava* EGI L10123^T^, that belongs to a new family *Salinilacustritrichaceae* fam. nov. We provide evidence of sulfide-dependent chemolithoautotrophy via the roTCA in this taxon. Moreover, ancestral character state reconstructions of key enzymes in *Acidimicrobiia* hint at the possibility of CO_2_ fixation via the roTCA.

## Materials and methods

### Sample collection and chemolithoautotrophic enrichment

**Figure 1:**
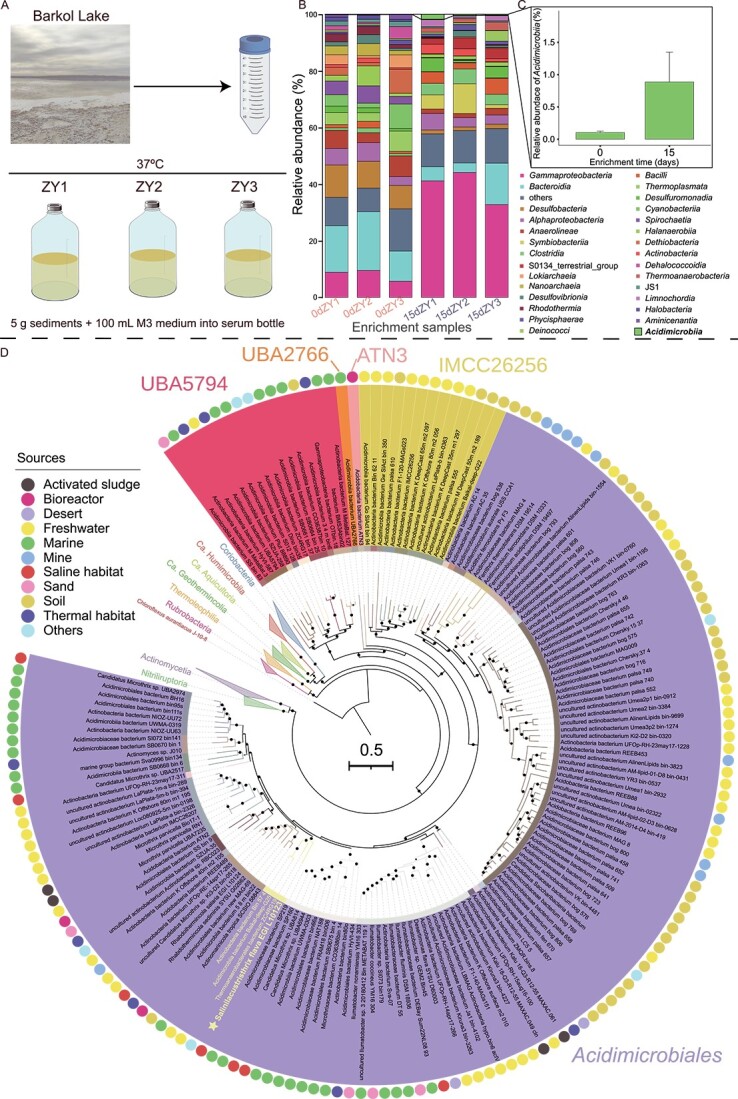
***Acidimicrobiia* enrichments and diversity in diverse environments.** (**A**) Schematic representation of the enrichment strategy for chemolithoautotrophic microorganisms in the saline lake. (**B**) Analysis of the community compositions of three original sediment samples from the saline lake and samples after chemolithoautotrophic enrichment. (**C**) Comparative assessment of the relative abundance of *Acidimicrobiia* pre- and post-chemolithoautotrophic enrichment (error bar represents standard deviation). (**D**) Phylogenetic analysis of *Acidimicrobiia*. The maximum-likelihood phylogenetic tree was constructed using multiple sequence alignments of 120 bacterial marker genes generated by GTDB-Tk [20] according to IQ-Tree software [21]. The best-fit model LG + F + I + I + R10 was well-supported by Bayesian Information Criterion. Bootstrap values were based on 1000 replicates and nodes with percentages >90% indicated as solid circles. Circles outside the tree labels illustrate genome sources. The genomes with star was obtained from this study.

Surface sediment samples from a depth of ~20 cm were collected from a hypersaline lake, Barkol Lake (92°48′11″E, 43°40′05″N), in Xinjiang Province, PR China (Fig. S1). The sediment at the sampling site exhibits a total salt content of 207 g/kg and a pH of 8.9, as analyzed under the People’s Republic of China National Standard for Environmental Quality Standard for Soils (GB 15618-1995) at the Xinjiang Institute of Ecology and Geography, Chinese Academy of Sciences. Similarly, the water at the sampling site shows a salinity of 66 g/L and a pH of 7.9, as determined under the People’s Republic of China National Standard for Environmental Quality Standard for Surface Water (GB 3838-2002) at the same institute. Two samples of Barkol Lake were collected (one in 2020 and the other in 2021). Samples for DNA extraction were stored at −80°C in sterile sampling tubes and samples for laboratory enrichment were kept at 4°C in sterile sampling bags. To ensure the sediments were homogenized as much as possible, we continuously stirred the samples overnight before inoculation. Three replicates of chemolithoautotrophic enrichment cultures were constructed (Fig. 1A) by using a chemolithoautotrophic enrichment M3 medium (+V) containing a vitamin solution, trace elements, and Na_2_S as an electron donor (Table S1), with the Barkol Lake sample collected in 2020. The medium was dispensed in 100 mL volumes in 250 mL serum bottles covered in a vented sealing film, inoculated with 5 g of sediment, and incubated at 37°C under oxic conditions (atmospheric air). All glassware was rinsed twice with 6 M HCl, followed by three rinses with Milli-Q water, autoclaved, and dried at 60°C before use to remove any contaminants that could interfere with the experiments as thoroughly as possible [22].

### DNA extraction, sequencing, and analysis of 16S rRNA gene amplicons

DNA for high-throughput sequencing from the original sample and enrichment cultures was extracted using the FastDNA Spin Kit (MP Biomedicals). The integrity and concentration of the extracted DNA were checked by agarose gel electrophoresis and Invitrogen Qubit Fluorometer. Samples for amplicon sequencing were sent to Beijing Biomarker Technologies Co, Ltd for PCR amplification, library construction, and sequencing. The V4 region of the 16S rRNA gene was amplified using the primers 515F (5’-GTGCCAGCMGCCGCGGTAA-3′) and 806R (5’-GGACTACHVGGGTWTCTAAT-3′) [23]. Amplicons were then sequenced using the NovaSeq platform (Illumina, USA). The raw amplicon reads were processed using the cutadapt plugin with parameters —p-front-f GTGCCAGCMGCCGCGGTAA —p-front-r GGACTACHVGGGTWTCTAAT in the QIIME2 (ver. 2023.2) platform to remove the primers [24]. Then, the DADA2 plugin in the QIIME2 platform was used to merge, denoise, filter chimeras, dereplicate, and obtain feature tables as well as representative amplicon sequence variant (ASV) sequences [25]. Each ASV was classified using the qiime feature-classifier plugin based on the SILVA database (Release 138.1) [26–28].

### Metagenomic sequencing and analysis

For metagenomic sequencing, DNA samples were prepared using a VAHTS Universal Plus DNA Library Prep Kit for Illumina, and the insert size was assessed using Agilent 2100. Metagenomic sequencing was performed using the NovaSeq platform with PE150 (Illumina, USA) at Beijing Biomarker Technologies Co, Ltd. Approximately 30 Gbp of metagenomic data for each sample was generated (Table S1). The raw metagenomic reads were quality filtered to eliminate adapters, duplicated reads, and low-quality reads by using fastp (ver. 0.23.4) with parameters -q 20 -u 20 -e 20 -l 50 -5 -3 --cut_front_window_size 50 --cut_front_mean_quality 20 --cut_tail_window_size 50 --cut_tail_mean_quality 20 -w 20 [29, 30]. High-quality reads were de novo assembled into scaffolds using SPAdes (ver. 3.15.5) with parameters --meta -k 21,33,55,77,99 [31]. Genome binning was performed by using MetaBAT2 with the scaffolds >2500 bp [32]. CheckM (ver. 1.2.2) was used to analyze the completeness and contamination of the MAGs [33]. MAGs with estimated completeness <80% or contamination >5% were discarded. MAGs were taxonomically classified using GTDB-Tk (ver. 2.3.2) [20]. Protein-coding regions were predicted using Prodigal (ver. 2.6.3) [34]. MAGs were further annotated by querying the predicted coding sequence (CDS) against the Clusters of Orthologous Groups (COG) [35] as well as Kyoto Encyclopedia of Genes and Genomes (KEGG) databases (Release 108.1) using DIAMOND (ver. 0.7.9; E-values <1e-5) [36, 37].

### Isolation and identification of a new chemolithoautotrophic *Acidimicrobiia* family

We employed a culture-dependent method [38, 39] to selectively isolate *Acidimicrobiia* from both the enrichment culture and the original sediment samples using nutrient medium (0.5 × Marine Agar 2216E, pH = 7.6, BD Difco™) as well as the chemolithoautotrophic M3 medium (+V, pH = 7.8) supplemented with 20% agar under oxic conditions (atmospheric air). For sediments, 1 g was added to 9 mL of sterile water, diluted to 10^−3^ in sterile water, and then spread onto plates for isolation. For the enrichment sample, 1 mL enrichment sample was added to 9 mL of sterile water, diluted to 10^−3^ in sterile water, and then spread onto plates for isolation. Pure cultures were obtained by repeating streaking of isolated colonies. Isolates were stored in a 20% glycerol solution at −80°C [39] and identified by amplifying near-complete 16S rRNA genes using primers (27F: 5’-AGAGTTTGATCCTGGCTCAG-3′ and 1492R: 5’-TACGACTTAACCCCAATCGC-3′) [40] followed by sequencing at Sangon Biotech (Shanghai) Co, Ltd. 16S rRNA gene sequences were analyzed using EzBioCloud (Release Note 20230823, https://www.ezbiocloud.net/). From these efforts, a single new isolate belonging to *Acidimicrobiia* was successfully obtained. This isolate has been officially designated as strain EGI L10123^T^. A polyphasic taxonomy approach [41] was conducted as described in the Supplementary Information. To assess chemolithoautotrophic growth of strain EGI L10123^T^ under oxic conditions, a 1 mL cell suspension was inoculated into 250 mL sterile conical flasks under atmospheric air, each equipped with a vented sealing film, and filled with 100 mL of M3 medium (+V) or 100 mL of M3 medium deficient in vitamins (-V) using bicarbonate as the carbon source (Table S1), sulfide as the electron donor, and oxygen (from air) as an electron acceptor. To assess the chemolithoautotrophic growth potential of strain EGI L10123^T^ under anoxic conditions with N_2_ headspace gas, a 1 mL cell suspension was inoculated into 250 mL sterile serum bottles and filled with 100 mL of M3 medium (+V) or 100 mL of M3 medium deficient in vitamins (-V) using bicarbonate as the carbon source (Table S1). After consecutive transfers in M3 medium (-V), the number of cells was measured by flow cytometry to assess chemolithoautotrophic growth.

We also quantitatively assessed the enzymatic activity of citrate synthase (CS) in strain EGI L10123^T^ under chemolithoautotrophic conditions in M3 medium (-V) at 37°C for 12 days using the CS activity assay kit (Beijing Solarbio Science & Technology Co, Ltd). The sulfide-quinone oxidoreductase (SQR) enzyme was also quantified in strain EGI L10123^T^ using an enzyme-linked immunosorbent assay (ELISA) kit for SQR from Jiangsu Yutong Biotechnology Co, Ltd, during cultivation in 100 mL of chemolithoautotrophic M3 medium (-V) at 37°C for 6 and 12 days.

### Complete genome sequencing and analysis of EGI L10123^T^

Genomic DNA was extracted using the TIANamp Bacteria DNA Kit and sent to Guangdong Magigene Biotechnology Co Ltd for Illumina short-read sequencing as well as Beijing Biomarker Technologies Co, Ltd for PacBio long-read sequencing. The complete genome of strain EGI L10123^T^ was assembled using Unicycler software (ver. 0.5.0) based on a hybrid assembly [42]. Protein-coding regions were predicted using Prodigal (ver. 2.6.3) [34]. Functional annotation of EGI L10123^T^ was also performed by querying predicted CDS against the KEGG database (Release 108.1) using DIAMOND (ver. 0.7.9; E-value <1e^−5^) [36, 37].

### Transcriptome sequencing and analysis of EGI L10123^T^ grown under chemolithoautotrophic conditions

For transcriptome sequencing, EGI L10123^T^ cells were grown in 10 L of M3 (-V) medium with bicarbonate as the sole carbon source, sulfide as the sole electron donor, oxygen (from air) as the electron acceptor, and harvested by filtration (0.22 μm membrane). Filters were transferred immediately to liquid nitrogen and stored at −80°C. Total RNA was extracted using the QIAGEN RNeasy PowerSoil Total RNA Kit (MOBIO). Quality checking, genomic DNA digestion, ribosomal RNA removal, cDNA synthesis, and library construction were performed, and transcriptome data were sequenced by using the NovaSeq 6000 (Illumina) instrument with PE150 at Novogene. Raw transcriptome data were pre-processed as described for the genomic data, and rRNA sequences were removed by using SortMeRNA (ver. 4.3.6) [43]. Finally, the filtered transcriptomic reads were mapped to strain EGI L10123^T^ genes by Salmon (ver. 1.10.1) [44].

### Stable isotope evaluation of the carbon fixation pathway in strain EGI L10123^T^

A ^13^C-labeling experiment was conducted to detect isotopologues of intermediate metabolites to determine the specific carbon fixation pathway in the chemolithoautotrophic M3 medium (-V) containing 200 g/L NaH^13^CO_3_ as the sole carbon source, sulfide (Na_2_S) as the sole electron donor, and oxygen (from air) as an electron acceptor under oxic conditions (under normal atmospheric pressure conditions). Cultivation was conducted in 30 1000 mL conical flasks (each bottle containing 500 mL medium, in total 15 L) with 150 rpm agitation at its optimal growth temperature of 37°C, using strain EGI L10123^T^ after two consecutive rounds of transfer growth in M3 medium (-V). All conical flasks were rinsed twice with 6 M HCl, followed by three rinses with Milli-Q water, autoclaved, and dried at 60°C before use. The cells were harvested by filtration through a 0.22 μm membrane after 7 days of incubation for isotopologue profiling based on liquid chromatography-mass spectrometry (LC–MS) analysis. The method for LC–MS analysis and calculation of excess ^13^C were done as described previously [45, 46]. Glutamate, aspartate, and serine were selected for the isotopologue analysis because they are synthesized from the precursors generated by the roTCA [45]. Although alanine is another roTCA indicator, the EGI L10123^T^ strain is missing the alanine aminotransferase (ALT) gene, which means it cannot synthesize alanine from pyruvate and therefore ^13^C-labeled alanine would not be expected as an intermediate.

### Phylogenetic analysis

The complete genome of strain EGI L10123^T^ together with a reference actinobacterial genomic dataset [14] and 167 high-quality *Acidimicrobiia* genomes (completeness >95% or contamination <5%) from the GTDB database (Release 214.1) were used for phylogenetic analysis to determine the phylogenetic position of strain EGI L10123^T^. A maximum-likelihood phylogenetic tree was constructed using a multiple sequence alignment of 120 bacterial marker genes generated by GTDB-Tk (ver. 2.3.0) [20] by using IQ-Tree software (ver. 2.2.2.7) [21] with parameters (−alrt 1000 -bb 1000 AUTO). The best-fit model (LG + F + I + I + R10) was well-supported by the Bayesian Information Criterion (BIC).

For phylogenetic analysis of proteins of interest, datasets were derived from the literature and the NCBI prokaryotic gene database. Reference amino acid sequences for ribulose-1,5-bisphosphate carboxylase/oxygenase (RuBisCO) large subunit (RbcL) and NiFe hydrogenases (group1b, 1g, 1h, 2a, 3b, 3d, 4b, 4f) were obtained from previous studies [47–49]. Protein sequences were aligned using MUSCLE (ver. 3.8.31) [50] with 100 iterations, and divergent regions were removed using TrimAL (ver. 1.4) [51]. Phylogenetic inference was performed using IQ-Tree (ver. 2.2.2.7) [22] with parameters (−alrt 1000 -bb 1000 AUTO) and the best-fit model (RbcL: LG + F + R10, [NiFe] hydrogenases groups 1, 2, and 3: LG + R10, as well as [NiFe] hydrogenases group 4: VT + F + R10). The chosen model was well-supported by the BIC. All trees were visualized and annotated using Interaction Tree of Life (iTOL, https://itol.embl.de/).

The evolutionary history of *Acidimicrobiia* was inferred by using COUNT [52] as previously described [14, 53, 54]. MrBayes (ver. 3.2.0) was used for constructing the Bayesian tree with parameters (ngen = 2 000 000 Nruns = 2 Nchains = 4 diagnfreq = 1000 relburnin = yes burninfrac = 0.25 261 samplefreq = 100 printfreq = 100) using the multiple sequence alignment of 120 bacterial marker genes generated by GTDB-Tk (ver. 2.3.0). The results of the standard deviation of split frequencies (<0.05), the potential scale reduction factor (PSRF = 1), and the effective sample size (ESS > 100) made the Bayesian tree highly reliable. The time-calibrated phylogenetic tree was further inferred by using R (http://cran.r-project.org/, ver. 4.3.1) with the ape package. All trees were visualized and annotated by using iTOL (https://itol.embl.de/).

### Distribution and abundance of *Acidimicrobiia*

Full-length 16S rRNA gene sequences from each *Acidimicrobiia* genome were submitted to the IMNGS online server (https://www.imngs.org/) [55] for searching against all 16S rRNA gene amplicon datasets from the NCBI Sequence Read Archive (SRA), using a minimum identity threshold of 99%. Samples were removed if the relative abundance of target sequences was <0.1%. Global distribution display was carried out in the R program (http://cran.r-project.org/, ver. 4.3.1).

## Results and discussion

### Relative abundance of *Acidimicrobiia* increased after chemolithoautotrophic enrichment

Chemolithoautotrophic microorganisms are believed to contribute significantly to primary production within saline lakes [56]. To study aerobic chemolithoautotrophs in this environment (Fig. S1), oxic enrichments were set up using bicarbonate as the sole carbon source, sulfide as the electron donor, and Barkol Lake sediments as the inoculum (Fig. 1A). Following a 15-day enrichment at 37°C, 16S rRNA gene amplicon (V4 region) analysis revealed changes in the microbial community composition compared to the original sediment samples (Fig. 1B). We found that *Acidimicrobiia*, which initially had a low relative abundance (day 0), increased following chemolithoautotrophic enrichment (day 15) (Fig. 1C). It has been previously suggested that some *Acidimicrobiia* possess the potential for carbon fixation via the CBB cycle based on the presence of genes encoding hydrogenase and RuBisCO enzymes (including oxygen-producing photoautotrophic RuBisCO type IA and chemoautotrophic RuBisCO type IE) in several *Acidimicrobiia* MAGs [14–16, 18, 19], but to our knowledge autotrophy has never been experimentally verified in *Acidimicrobiia*. However, our analysis of three high-quality *Acidimicrobiia* MAGs obtained from the sediments failed to uncover key genes (*rbcL*, *rbcS*, *prk*) [57] associated with the CBB cycle, or other intact gene clusters involved in other well-known carbon fixation pathways (Fig. S2 and Table S2). Therefore, we sought to determine whether *Acidimicrobiia* in general or the specific strains have the capability to be chemolithoautotrophic.

### Isolation and identification of a new chemolithoautotrophic *Acidimicrobiia* family

**Figure 2:**
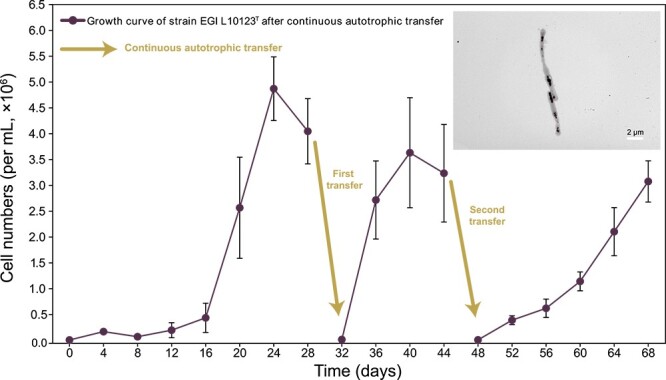
**Verification of chemolithoautotrophy of strain EGI L10123** ^**T**^ **.** Transmission Electron Micrograph (TEM) cell morphology photos of strain *Salinilacustrithrix flava* EGI L10123^T^ is shown in the upper right corner of the figure. Cells of strain EGI L0123^T^ were long rod-shaped, each about 5.0–10.0 μm long and 0.4–0.5 μm wide. Error bar represents standard deviation.

We performed a culture-dependent strategy [38, 39] to isolate *Acidimicrobiia* from both the enrichment culture and the original sediment samples. This strategy involved the use of a nutrient medium (0.5 × Marine Agar, 2216E), in combination with a chemolithoautotrophic M3 medium (+V) containing sulfide as the electron donor and bicarbonate as the sole carbon source, both supplemented with 20% agar. A new *Acidimicrobiia* strain EGI L10123^T^ was isolated from the original sample after 15 days of incubation using a nutrient medium (0.5 × Marine Agar, 2216E) and subsequent repeated colony isolation. Negative staining under transmission electron microscope (TEM) showed that cells of strain EGI L10123^T^ were long rod-shaped (0.4–0.5 μm wide and 5.0–10.0 μm long) (Fig. 2). Moreover, strain EGI L10123^T^ grew at 28–37°C (optimum 37°C), pH 6.0–10.0 (optimum pH 8.0), and in the presence of 0–5% (w/v) NaCl (optimum 0%) using a nutrient medium (Marine Agar, 2216E) (Table S3). These traits are consistent with its environmental source in the saline lake sediment. The complete genome of strain EGI L10123^T^ was obtained and consisted of a single contig 4 107 660 bp bases long, encoding 3931 predicted CDSs, 3 rRNAs (one 23S rRNA, one 16S rRNA, and one 5S rRNA), and 51 tRNAs. The EGI L10123^T^ genome, along with 167 publicly available high-quality *Acidimicrobiia* MAGs from a variety of environments (Fig. 1D), were analyzed to further assess their diversity, physiology, and evolution. The phylogenetic position and distinctness of EGI L10123^T^ based on relative evolutionary divergence (0.3163809616) indicated that EGI L10123^T^ is a new species of a new family (d__*Bacteria*; p__*Actinobacteriota*; c__*Acidimicrobiia*; o__*Acidimicrobiales*; f__JACDCH01; g__; s__) within the order *Acidimicrobiales* (Fig. 1D and Table S4). Here, we propose the name *Salinilacustristhrix flava* EGI L10123^T^ as the pure culture of the new family *Salinilacustritrichaceae*. For a comprehensive analysis of the taxonomy and description of strain EGI L10123^T^, refer to Supplementary Information, Figs S3–S7, and Tables S5–S6. Although vitamins are typically considered growth factors rather than carbon sources, to exclude the possibility of growth on trace organic compounds in the vitamin solution, cultivation was also conducted in a M3 medium without the addition of trace vitamins. Therefore, chemolithoautotrophic growth of strain EGI L10123^T^ was confirmed by cultivating it in M3 medium with bicarbonate as the sole carbon source and sulfide as the sole electron donor, with (+V) or without trace vitamins (−V), under oxic and anoxic conditions. The results showed that the strain was able to grow in the presence/absence of vitamin solution under oxic conditions (Fig. S8). Subsequently, after three successive transfers, strain EGI L10123^T^ maintained growth in M3 medium (−V) under oxic conditions (atmospheric air, Fig. 2), showing that this new strain is capable of chemolithoautotrophy.

### Multi-omics insights into the roTCA-based carbon fixation by EGI L10123^T^

**Figure 3:**
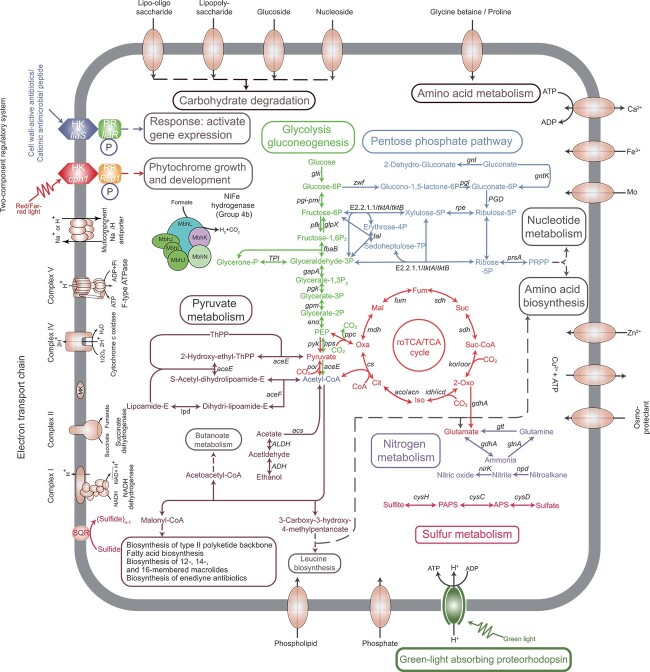
**Overview of metabolic capabilities of strain EGI L10123** ^**T**^ **.** Different module represents different metabolic modules. Cit: Citrate, Iso: Isocitrate, 2-Oxo: 2-Oxoglutarate, Succ-CoA: Succinyl-CoA, Succ: Succinate, Fum: Fumarate, Mal: Malate, Oxa: Oxaloacetate, PRPP: 5-Phospho-alpha-D-ribose 1-diphosphate, ThPP: Thiamine pyrophosphate, PEP: Phosphoenolpyruvate. This figure was generated according to the gene data in Table S7 using Adobe Illustrator 2022 software.

To gain insight into possible carbon fixation pathways and other metabolic features of strain EGI L10123^T^, we reconstructed major metabolic pathways (Fig. 3, Table S7, and Supplementary Information; Figs S9–S14). Previous studies suggested that some members of the class *Acidimicrobiia* are capable of carbon fixation through the CBB cycle, as determined through metagenomic analysis [15]. However, a comprehensive genome analysis of strain EGI L10123^T^ revealed the absence of key genes that are required for well-known chemolithoautotrophic pathways, including *rbcL*, *rbcS*, and *prk* of the CBB cycle [15, 58]. These genes were also sparse across 168 high-quality *Acidimicrobiia* genomes, suggesting that the CBB cycle is not a prevalent carbon fixation pathway within this class (Fig. S15). A phylogenetic tree of the 1,5-ribulose bisphosphate carboxylase (RbcL) was constructed to investigate the evolution of this protein in the class *Acidimicrobiia* (Fig. S16). This revealed a sparse distribution of *Acidimicrobiia* RbcL homologs across two distinct forms: Form I (A and E), which is responsible for oxygenic photoautotrophs and chemoautotrophs via the CBB cycle, and Form IV RuBisCO-like proteins (RLP), which are not known to catalyze ribulose 1,5-bisphosphate-dependent CO_2_ fixation [47]. These results implied that the presence of the *rbcL* gene in this class was due to multiple horizontal gene transfers from distinct microbial taxa. This result suggests that the CBB cycle in this class is not a prevalent carbon fixation pathway. The absence of the CBB cycle in the EGI L10123^T^ genome implies that the other carbon fixation pathways may be functioning under the chemolithoautotrophic condition.

Despite the absence of key genes for well-known chemolithoautotrophic pathways, we noticed that EGI L10123^T^ encodes CS (*cs*, *gltA*) as well as the complete TCA cycle, suggesting that the reversed oxidative TCA (roTCA) may be functioning based on recent research findings that documented the reversibility of CS reactions for carbon fixation [45, 59, 60] (Fig. 3 and Table S7). Transcriptomic analysis of strain EGI L10123^T^ following growth on M3 medium lacking vitamins (−V) with bicarbonate as the sole carbon source and sulfide as the sole electron donor revealed multiple genes associated with the roTCA in the top 30 most abundant transcripts (Tables S8 and S9), including pyruvate ferredoxin oxidoreductase (POR), 2-oxoglutarate ferredoxin oxidoreductase (OGOR), and lower amounts of all other transcripts related to the roTCA (Tables S8 and S9). We also detected the activity of CS (116.6 U/g; U/g is defined as the amount of enzyme that catalyzes the production of 1 nmol of 5-thio-2-nitrobenzoic acid per minute per gram of cells in the reaction system) in strain EGI L10123^T^ grown under chemolithoautotrophic conditions in M3 medium (−V) at 37°C. Together these results implied that strain EGI L10123^T^ may employ the roTCA to fix CO_2_ [45, 59, 60].

### SIP-metabolomics supported involvement of the roTCA in carbon fixation of strain EGI L10123^T^

**Figure 4:**
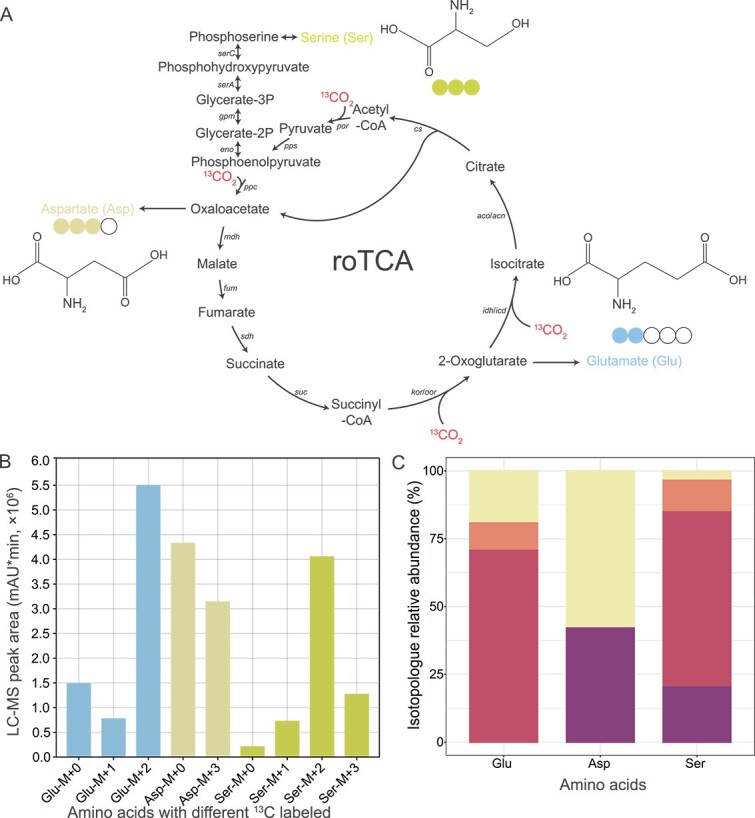
**Analysis of labeling experiments to track the roTCA activity.** (**A**) The incorporation of ^13^CO_2_ into serine, aspartate, and glutamate after several rounds of the roTCA produces diverse isotopologues. (**B**) LC–MS peak area (mAU*min) of the detected isotopologues. (**C**) The relative abundances of the detected isotopologues. The labeled carbons are schematically represented as circles, in accordance with the legend on the histogram plot. M + X refers to isotopologues carrying X ^13^C atoms.

To test for carbon fixation via the roTCA in strain EGI L10123^T^, SIP-metabolomics was carried out using cells grown chemolithoautotrophically with a high concentration (200 g/L) of NaH^13^CO_3_ as the sole carbon source and sulfide as the electron donor. Cells were harvested during late-exponential growth phase, and the labeling pattern of proteinogenic amino acids was examined using LC–MS. The number of ^13^C-labeled carbon atoms in the three amino acids, glutamate, aspartate, and serine were measured as described previously [45], which are direct products of the TCA cycle intermediates 2-oxoglutarate, oxaloacetate, and phosphoenolpyruvate (Fig. 4A and Table S10). The results showed a high amount of ^13^C-labeled (M + 1) glutamate (10.07%), (M + 2) glutamate (70.72%), (M + 3) aspartate (42.08%), (M + 1) serine (11.67%), (M + 2) serine (64.54%), and (M + 3) serine (20.32%) in NaH^13^CO_3_-grown cells. These results provide unequivocal evidence of a functional roTCA in strain EGI L10123^T^ (Fig. 4B and C). The presence of isotopologues indicates ongoing carbon fixation, indicative of the assimilation of ^13^CO_2_, whereas heavier isotopologues (≥M + 3) indicate carbon fixation during multiple rounds of the roTCA. The initial reaction of the roTCA is catalyzed by CS, converting citrate into acetyl-CoA and oxaloacetate. Moreover, previous studies have suggested that the detection of aspartate molecules labeled with three or four ^13^C indicates that oxaloacetate undergoes multiple cycles in the reductive tricarboxylic acid (roTCA) [59]. Although heavy isotopologues (M ≥ 4) were not detected in this study, possibly due to limited detection or interference with other metabolites, the growth of strain EGI L10123^T^ under chemolithoautotrophic conditions, the high expression of key roTCA genes (*ppc*, *por*, and *kor*) (Table S9), and the detection of other key isotopologues together support the possibility of the roTCA being involved in carbon fixation of strain EGI L10123^T^.

### Energy metabolism in strain EGI L10123^T^

Due to the substantial energy and reducing equivalents required by chemolithoautotrophic bacteria for synthesizing organic compounds from inorganic carbon and ATP synthesis, electron donors can become a critical growth-limiting factor [61, 62]. Chemolithoautotrophic prokaryotes rely on inorganic electron donors to provide reducing power for electron transport and ATP synthesis. Given that sulfide was the electron donor in M3 medium (−V), we inferred a role for the SQR, which catalyzes the oxidation of sulfide to sulfite and thiosulfate, in chemolithoautotrophic growth of strain EGI L10123^T^ (Fig. 3). Sulfur quinone oxidoreductase, located on the bacterial cell membrane, catalyzes the oxidation of sulfides into sulfates and sulfite, producing an excess of electrons [63]. These electrons are then transmitted into the electron transport chain, providing ample energy for chemolithoautotrophic carbon fixation [64]. Transcriptomic analysis of strain EGI L10123^T^ under chemolithoautotrophic conditions revealed high expression levels of the *sqr* gene and electron transport chain genes, including the terminal reductase, cytochrome c oxidase (*COX10*, *COX15*, *coxA*, *coxB*, and *cyoC*), and an F_o_F_1_-type ATP synthase (Tables S8 and S9). To further probe the expression of SQR, an ELISA was used to quantify SQR proteins in crude extracts from strain EGI L10123^T^ following cultivation in chemolithoautotrophic M3 medium (−V) at 37°C for 6 and 12 days. The results revealed 15 and 25 ng/100 mL, demonstrating the ability of strain EGI L10123^T^ to both transcribe and translate the *sqr* gene under chemolithoautotrophic conditions (Fig. S17A). We also observed that the concentration of S^2−^ in the culture medium gradually decreased (Fig. S17B). Sulfide (S^2−^) oxidation by SQR produces various polysulfides (sulfide)_n-1_, including thiosulfate (S_2_O_3_^2−^). The detection results also showed an increase in thiosulfate (S_2_O_3_^2−^) concentration, and S_2_O_3_^2−^ was not detected in the negative control incubations (Fig. S17C). The above evidence further suggested sulfide oxidation, with thiosulfate being one of the primary products resulting from the sulfide oxidation. The transcriptomic analysis of strain EGI L10123^T^ also uncovered high levels of transcripts of key gluconeogenesis genes, including *fbp*, *glpX*, *pfk*, *ppdK*, and *pps* (Tables S8 and S9). Carbon fixation transforms carbon atoms from non-carbohydrate precursors into pyruvate or acetone, followed by gluconeogenesis synthesizing two pyruvate molecules into a hexose sugar, which is subsequently metabolized by glycolysis back into acetyl-CoA, providing energy [65, 66]. Together, these results show that chemolithoautotrophy via the roTCA in strain EGI L10123^T^ may be fueled by aerobic sulfide oxidation.

### Evolutionary history of chemolithoautotrophy in *Acidimicrobiia*

With evidence of a sulfide-dependent roTCA in strain EGI L10123^T^, we sought to better understand the distribution as well as evolution of the roTCA and chemolithotrophy in the *Acidimicrobiia*. The three key genes for the roTCA, CS, pyruvate:ferredoxin oxidoreductase (POR), and 2-oxoglutarate:ferredoxin oxidoreductase (OGOR) [45], were nearly universal in *Acidimicrobiia* (Fig. S18). In light of this finding, we speculate that nearly all members of the *Acidimicrobiia* have the potential to perform CO_2_ fixation through the roTCA rather than the CBB cycle. Although the thermodynamics of citrate cleavage by CS in the roTCA are unfavorable (free-energy change ∆G′ of more than 35 kJ mol^−1^) [67], it allows the synthesis of acetyl-CoA from the two molecules of CO_2_ with reduced ATP expenditure. This characteristic makes the roTCA an energetically efficient chemolithoautotrophic carbon fixation pathway [45, 60]. Additionally, the roTCA pathway has been shown to fix CO_2_ in a variety of taxa by using on SIP-metabolomics. For example, the roTCA pathway has been demonstrated under high CO_2_ partial pressures in the acetate-oxidizing and sulfur-reducing *Campylobacterota* bacterium *Desulfurella acetivorans* [60], the hydrogen-oxidizing and sulfur-reducing *Thermosulfidibacterota* bacterium *Thermosulfidibacter takaii* [68], the Fe (III)-reducing *Desulfobacterota* species *Geobacter sulfurreducens* [59], the extremophilic nitrite-oxidizing *Chloroflexota* “*Candidatus* Nitrotheca patiens” [69], the Fe (III)-reducing *Pseudomonadota* bacterium *Deferribacter autotrophicus* [70], and the marine *Campylobacterota* species *Hippea maritima* [45]. The above-mentioned taxa are mainly anaerobic microorganisms, whereas in this study, strain EGI L10123^T^ operates carbon fixation via the roTCA in an exceptionally high bicarbonate medium under oxic conditions. And, we have also detected the highest expression (Transcripts per million, TPM = 5185.286592) of the carbonic anhydrase (Table S8). Carbonic anhydrase dehydrates HCO_3_^−^ to CO_2_ provides high levels of CO_2_ for POR and OGOR for producing oxo-acids. The concentration of CO_2_ obtained via carbonic anhydrase makes sense in light of the endergonic nature of carbon fixation using these enzymes. This discovery suggests that roTCA may operate both aerobically and anaerobically in environments with high concentrations of bicarbonate or CO_2_, potentially expanding the habitat range of roTCA functionality.

The utilization of inorganic carbon by chemolithoautotrophic bacteria for the synthesis of organic compounds necessitates a substantial expenditure of energy and reducing equivalents. This process can also help to remediate greenhouse gas emissions by converting inorganic carbon into organic carbon in diverse ecosystems. Inorganic electron donors, such as hydrogen (H_2_), reduced nitrogen (NH_4_^+^ and NO_2_^−^), and reduced sulfur (e.g. S_2_^−^, S^0^, and S_2_O_3_^2−^), serve as important sources of reducing power for chemolithoautotrophic metabolism [71]. Hydrogen is recognized as a near-universal electron donor and almost all *Acidimicrobiia* genomes were predicted to encode hydrogenases (Fig. S19). NiFe hydrogenase groups 3b and 4b were most common in the *Acidimicrobiia* genomes (Figs S20 and S21). The wide distribution and monophyly of group 4b NiFe hydrogenases from *Acidimicrobiia* suggest that these genes were likely present in the common ancestor of the class *Acidimicrobiia* and extended by vertical inheritance. Thus, most *Acidimicrobiia* may use group 4b NiFe hydrogenases to oxidize formate or carbon monoxide while reducing protons to generate a sodium-motive force through Mrp antiporter modules [48], which is essential for the growth of a variety of halophilic and alkaliphilic bacteria under stressful conditions [72]. Group 3b NiFe hydrogenases are also common and widely distributed in *Acidimicrobiia*, although they are not monophyletic. These hydrogenases may catalyze NADPH oxidation and the fermentative evolution of H_2_ [48]. Furthermore, most *Acidimicrobiia* also encode SQR (Fig. S22), suggesting sulfide as a near-universal electron donor in *Acidimicrobiia*, although SQR is also known to be used for sulfide detoxification [63, 73]. According to existing research, despite the recognized toxicity of sulfide, it holds metabolic significance as an electron donor for both chemotrophic and photosynthetic organisms [58]. Sulfide quinone oxidoreductase (SQR) oxidizes sulfide into polysulfides and transfers electrons to the electron transport chain of aerobic respiration, generating the proton motive force essential for ATP production [59]. Given the widespread presence of diverse hydrogenases and SQR within the *Acidimicrobiia*, we suggest that hydrogen and sulfide may be key electron donors for carbon fixation in *Acidimicrobiia*.

**Figure 5:**
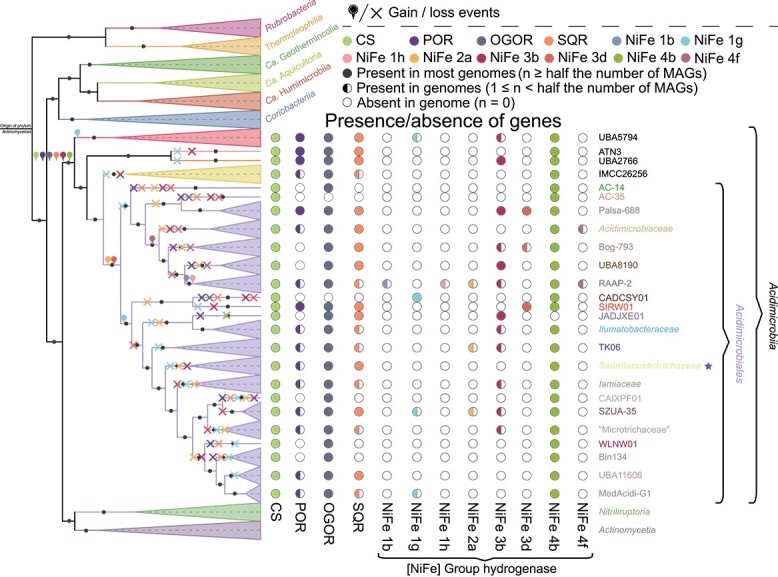
**Evolutionary history of chemolithoautotrophic function in *Acidimicrobiia*.** The Bayesian tree topology was established using MrBayes [74], employing multiple sequence alignments of 120 bacterial marker genes generated via GTDB-Tk [20]. The Bayesian tree’s robustness was substantiated by the convergence of key indicators: standard deviation of split frequencies (<0.05), PSRF (PSRF = 1), and ESS (ESS > 100). For time-calibrated phylogenetic inference, the R programming language was employed in conjunction with the ape package. The evolutionary trajectory of *Acidimirobiia* was deduced using COUNT [52], following established methodologies [14, 53], and corroborated via phylogenetic trees constructed for each protein dataset. Star denotes the newly proposed family *Salinilacustritrichaceae*, within which *Salinilacustrithrix flava* EGI L10123^T^ is situated.

**Figure 6:**
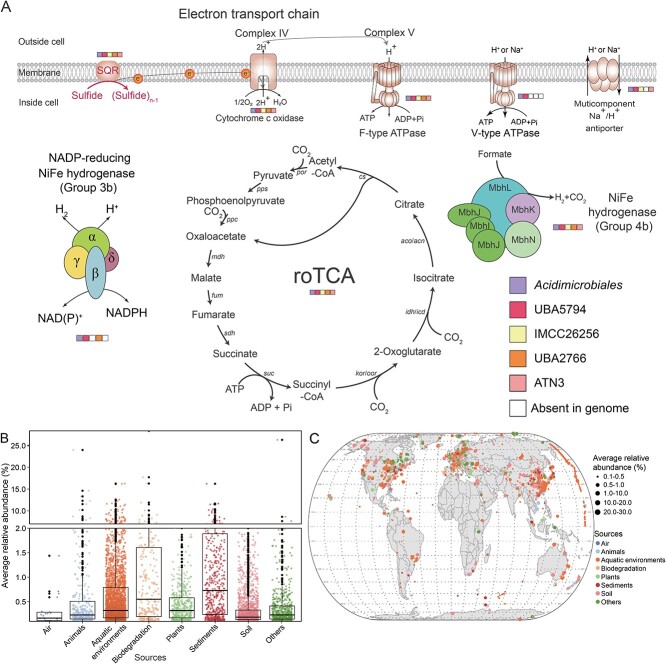
**Hydrogen- or sulfide-dependent roTCA and distribution of *Acidimicrobiia*.** (**A**) Model of the hydrogen- or sulfide-dependent roTCA in *Acidimicrobiia*. (**B**) Average relative abundance of *Acidimicrobiia* in different sample types. (**C**) Global distribution of *Acidimicrobiia* based on 8349 16S rRNA gene amplicon datasets in which *Acidimicrobiia* members were present, as a query result from the IMNGS platform [55]. *cs*: CS gene, *por*: POR gene, *pps*: pyruvate, water dikinase gene, *ppc*: phosphoenolpyruvate carboxylase gene, *mdh*: malate dehydrogenase gene, *fum*: fumarate hydratase gene, *sdh*: succinate dehydrogenase gene, *suc*: succinyl-CoA synthetase gene, *kor*/*oor*: 2-oxoglutarate/2-oxoacid ferredoxin oxidoreductase gene, *idh*/*icd*: isocitrate dehydrogenase gene, *aco*/*acn*: aconitate hydratase gene.

Ancestral character state reconstruction via COUNT was further used to gain insight into the evolution of key roTCA genes, SQR, and diverse hydrogenases across the evolutionary trajectory of *Acidimicrobiia* (Fig. 5). This analysis indicated that ancestral *Acidimicrobiia* acquired central metabolic functions related to the roTCA, sulfide oxidation, and hydrogen metabolism before the divergence of five of the orders (*Acidimicrobiales*, IMCC26256, ATN3, UBA2766, and UBA5794). To test the hypothesis that some of these other *Acidimicrobiia* can also grow chemolithoautotrophically, we chose three additional *Acidimicrobiia* strains. These strains were specifically chosen because they lack the CBB cycle and other carbon fixation pathways while possessing all the requisite genes associated with the roTCA (Table S7). These strains were *Desertimonas flava* DSM 149021^T^, *Actinomarinicola tropica* SCSIO 58843^T^, and *Rhabdothermincola sediminis* SYSU G02662^T^. Our experiments demonstrate that these strains can indeed grow under chemolithoautotrophic conditions, regardless of the presence or absence of trace vitamins (using M3 medium with a high concentration (200 g/L) of NaH^13^CO_3_ as the sole carbon source and sulfide as the electron donor under normal atmospheric pressure conditions). This is evidenced by a substantial increase in cell numbers observed (Fig. S23). Considering that the key genes for chemolithoautotrophy are ubiquitous in the class *Acidimicrobiia*, and that other *Acidimicrobiia* strains lacking the CBB pathway are also capable of chemolithoautotrophy, we speculate that the roTCA, SQR, and diverse hydrogenase constitute integral components of the potential metabolism of chemolithoautotrophy in *Acidimicrobiia* and may play a pivotal role in its ecological adaptability (Fig. 6A). Our results suggest that *Acidimicrobiia* thriving in eutrophic environments may have lost their ability to fix CO_2_ over the long term, leading to an overlook of their potential for chemolithoautotrophic carbon fixation. However, should environmental conditions change drastically, such as through a significant reduction in organic matter, their carbon fixation potential might be retrained and reactivated turning this capability into a crucial one. Further research is needed in the future to determine if these characteristics are actually widespread across the clade.

### Globally distribution traits of *Acidimicrobiia*

The isolation of EGI L10123^T^ and evidence for chemolithoautotrophy described here prompted us to explore the environmental and geographic distribution of chemolithoautotrophic *Acidimicrobiia*. A total of 94 nearly complete *Acidimicrobiia* 16S rRNA gene sequences were submitted to the IMNGS platform [55]. In total, 8349 16S rRNA gene amplicon datasets harbored operational taxonomic units with ≥99% similarity to *Acidimicrobiia* reference sequences, with highest prevalence and relative abundance in aquatic environments as well as sediments (Fig. 6B and C). Considering the evidence of prevalent chemolithoautotrophy in *Acidimicrobiia* and their widespread distribution in diverse ecosystems, our results imply that *Acidimicrobiia* could be important, yet underestimated chemolithoautotrophs.

## Conclusions

This study provides deep insight into the diversity of *Acidimicrobiia* as well as the potential of roTCA operating in *Acidimicrobiia*, driven by sulfide oxidation and probably hydrogen oxidation. Our evidence is based on a combination of genomics, growth experiments, transcriptomics, SIP-metabolomics, and phylogenetics and ancestral state reconstructions. Our study challenges traditional views on carbon fixation within this clade, which previously implied only the CBB based on metagenomes encoding key enzymes of the CBB. Additionally, our discovery may extend the occurrence of the roTCA both phylogenetically and ecologically by showing that the roTCA possibly operates both aerobically and anaerobically in environments with high concentrations of bicarbonate or CO_2_. This finding improves our understanding of the distribution of the roTCA and its ecological significance.

## Protologues

### Description of *Salinilacustritrichaceae* fam. nov. (ICNP)

*Salinilacustritrichaceae* (Sa.li.ni.la.cus.tri.thri.cha.ce’ae. N.L. fem. n. *Salinilacustrithrix*, the type genus of the family; −*aceae*, ending to denote a family; N.L. fem. pl. n. *Salinilacustritrichaceae*, the *Salinilacustrithrix* family). The description is the same as for the genus *Salinilacustristhrix*. The family contains the type genus *Salinilacustristhrix*.

### Description of *Salinilacustristhrix* gen. nov. (ICNP)

*Salinilacustrithrix* (Sa.li.ni.la.cus’tri.thrix. N.L. masc. adj. *salinus*, saline; N.L. masc. adj. *lacustris*, belonging to a lake; Gr. fem. n. *thrix*, a hair; N.L. fem. n. *Salinilacustrithrix*, a hair from a saline lake). The genus description at present is the same as the description of the type species, *Salinilacustrithrix flava*.

### Description of *Salinilacustrithrix flava* sp. nov. (ICNP)

*Salinilacustrithrix flava* (fla’va. L. fem. adj. *flava*, yellow, referring to the color of the colonies).

Cells are long rod-shaped, each about 5.0–10.0 μm long and 0.4–0.5 μm wide. Cells are not motile and have no flagella. Cells are Gram-stain positive and facultatively anaerobic. The major cellular fatty acids are Summed Feature 8 (C_17:1_  *ω*6*c* and/or C_17:1_  *ω*7*c*), iso-C_16:0_, anteiso-C_14:0_, and Summed Feature 3 (C_16:1_*ω*6*c* and/or C_16:1_*ω*7*c*). The G + C content of the genomic DNA is 71.81%. Cells contain menaquinones MK-9 (H_8_). Cells grow at pH values of 6 to 10, temperatures between 28 and 37°C, and NaCl concentrations between 0 and 5%. There is no growth under anoxic conditions without growth factors such as vitamins. The organism is positive for catalase, urease, milk peptonization and coagulation, gelatin liquefaction and coagulation, hydrolysis of cellulose, and degradation of Tweens (20, 40, 60, and 80), and oxidase, but starch negative. The type strain, EGI L10123^T^ (= CGMCC 1.19137^T^ = KCTC 49680^T^), was isolated from saline lake sediments.

## Supplementary Material

Tracked_Supplementary_Information_wrae147

Supplementary_Tables_wrae147

## Data Availability

The amplicon sequences generated in our study are available in the NCBI SRA database under the BioProject ID PRJNA1012838 with accession number SRR25905149 to SRR25905154. The MAGs described in this paper have been deposited in NCBI database under the BioProject ID PRJNA1012852. The GenBank accession numbers for the 16S rRNA gene sequence and the complete genome of strain EGI L10123^T^ are ON854140 and CP133888, respectively. The transcriptomic sequences of strain EGI L10123^T^ under the chemolithoautotrophic condition are available in the NCBI SRA database under the BioProject ID PRJNA1012866 with accession number SRR25905596.
