# Supplementary material for: Reversed oxidative TCA (roTCA) for carbon fixation by an Acidimicrobiia strain from a saline lake
Source: ISME J. 2024 Jul 29;18(1):wrae147. doi: 10.1093/ismejo/wrae147 (PMC11697166; doi:10.1093/ismejo/wrae147)
Supplement: Tracked_Supplementary_Information_wrae147 [file tracked_supplementary_information_wrae147.pdf]

Supplementary Information

~~Reversed oxidative TCA (roTCA)-operating carbon fixation in a new *Acidimicrobiia* strain~~Reversed oxidative TCA (roTCA) for carbon fixation by an *Acidimicrobiia* strain from a saline lake

Formatted: Font: Italic

Lei Gao[1,10†], Lan Liu[2†*], Ai-Ping Lv[2†], Lin Fu[3], Zheng-Han Lian[2], Takuro Nunoura[4], Brian P. Hedlund[5,6], Qing-Yu Xu[1,10], Dildar Wu[2], Jian Yang[7], Mukhtiar Ali[8], Meng-Meng Li[2], Yong-Hong Liu[1], André Antunes[9], Hong-Chen Jiang[1,7], Lei Cheng[3], Jian-Yu Jiao[2*], Wen-Jun Li[1,2*], Bao-Zhu Fang[1*]

[1]State Key Laboratory of Desert and Oasis Ecology, Key Laboratory of Ecological Safety and Sustainable Development in Arid Lands, Xinjiang Institute of Ecology and Geography, Chinese Academy of Sciences, Urumqi, 830011, PR China

[2]State Key Laboratory of Biocontrol, Guangdong Provincial Key Laboratory of Plant Resources and Southern Marine Science and Engineering Guangdong Laboratory (Zhuhai), School of Life Sciences, Sun Yat-Sen University, Guangzhou, 510275, PR China

[3]Key Laboratory of Development and Application of Rural Renewable Energy, Biogas Institute of Ministry of Agriculture and Rural Affairs, Chengdu, 610000, PR China

[4]Research and Development Center for Marine Biosciences, Japan Agency for Marine-Earth Science and Technology, Yokosuka, 237-0061, Japan

[5]School of Life Sciences, University of Nevada Las Vegas, Las Vegas, NV 89154, USA

[6]Nevada Institute of Personalized Medicine, University of Nevada Las Vegas, Las Vegas, NV 89154, USA

[7]Key Laboratory of Biogeology and Environmental Geology, China University of Geosciences, Wuhan, 430074, PR China

[8]Advanced Water Technology Laboratory, National University of Singapore (Suzhou) Research Institute, Suzhou, Jiangsu 215123, China

[9]State Key Laboratory of Lunar and Planetary Sciences, Macau University of Science and Technology, Taipa, Macau SAR, PR China

[10]University of Chinese Academy of Sciences, Beijing 100049, PR China

†These authors contributed equally to this work.

Correspondence:

Lan Liu, E-mail: liulan9@mail.sysu.edu.cn

Jian-Yu Jiao, E-mail: jiaojy5@mail.sysu.edu.cn
Wen-Jun Li, E-mail: liwenjun3@mail.sysu.edu.cn
Bao-Zhu Fang, E-mail: fangbaozhu@ms.xjb.ac.cn

Supplementary Text

Diversity of chemoautotrophic microbial taxa in hypersaline and oligotrophic lake ecosystems

171 representative high-quality MAGs were acquired based on metagenomic analysis for further identifying the chemoautotrophic microbial diversity and potential carbon fixation pathways within the chemoautotrophic enrichment culture and the original sample from the saline lake (Fig. S2 and Table S2). Consistent with the previous studies [1, 2], the presence of *rbcL*, *rbcS*, and *prk* in *Alphaproteobacteria* and *Gammaproteobacteria* is indicative of their ability to engage in carbon fixation via the CBB cycle. The presence of *por*/*nifJ*, *oorA*, and *aclB* in *Deinococci* is indicative of their ability to engage in carbon fixation via the rTCA cycle, which is consistent with previous reports [3]. Moreover, the presence of *fdh*, *fhs*, *folD*, *metF*, and *acsABCDE* in class *Desulfobacteria*, DSM-4660, and *Desulfovibrionia* of the phylum *Desulfobacterota* is indicative of their ability to engage in carbon fixation via the WLP, meanwhile a previously identified class *Ca*. Anaeroferrophilalia in this phylum also has potential WLP autotrophic capabilities [4].

Polyphasic taxonomy of strain EGI L10123^T

1. Phylogenetic analysis of strain EGI L10123^T

To determine the phylogenetic position of strain EGI L10123^T, the almost-complete 16S rRNA gene sequence of strain EGI L10123^T (1571 bp; accession number ON854140) was determined. Genomic DNA was extracted, and the 16S rRNA gene was PCR-amplified and sequenced as described by Fang et al [5]. Before sequencing, amplicons were purified by using a PCR purification kit (Sangon Biotech, China). After DNA extraction, PCR amplification, and 16S rRNA gene sequencing, the 16S rRNA gene sequences obtained were assembled using SeqMan program (DNAStar software ver. 17.1.1) and compared with the corresponding sequences of cultured species in the EzBioCloud database (Release Note 20230823, https://www.ezbiocloud.net/) [6] and the NCBI database by using BLAST search [7]. Comparative analyses of 16S rRNA gene sequences based on the EzBioCloud database indicated that strain EGI L10123^T was most closely related to *R. sediminis* SYSU G02662^T (92.04%), and the sequence similarity was near the family-level threshold value described by Konstantinidis et al [8], suggesting that this isolate might represent a new family in the order *Acidimicrobiales.* For phylogenetic analysis, the 16S rRNA gene sequences of closely related species were downloaded from GenBank and EzBioCloud databases and aligned using the Clustal W algorithm in MEGA X [9, 10]. Genetic distances were calculated using the Kimura 2-parameter model, and phylogenetic trees were constructed by the neighbor-joining, maximum-likelihood, and maximum-parsimony methods [11-14]. The maximum-likelihood phylogenetic tree based on 16S

rRNA gene sequences showed that EGI L10123^{T} was well-separated from other members of the order *Acidimicrobiales* (Fig. S3B). The stability of the phylogenetic tree was further confirmed by similar topologies of the neighbor-joining and maximum-parsimony trees (Fig. S4 and S5).

2. Genomic features of EGI L10123^{T} and related species

The complete genome of strain EGI L10123^{T} was assembled using Unicycler software (ver. 0.5.0) from Illumina short-read (MAGIGENE, Guangzhou, China) and PacBio long-read data (Biomarker, Beijing, China) [15]. Protein-coding regions in the assembled sequences were predicted using Prodigal (ver. 2.6.3) [16]. tRNA and rRNA were separately predicted by tRNA-scan (ver. 2.0.12) and RNAmmer (ver. 1.2), respectively [17, 18]. The functional annotation of EGI L10123^{T} was performed by querying the predicted CDS against the Clusters of Orthologous Groups (COG) [19] and Kyoto Encyclopedia of Genes and Genomes (KEGG) (Release 108.1) [20] databases using DIAMOND (ver. 0.7.9; E-values < $1e^{-5}$) [21]. The G + C content was calculated from the genome sequences. Transmembrane helices were predicted by TMHMM-2.0 [22]. The complete genome map was generated by GenoVi software (ver. 0.2.16) [23]. The genomic features of EGI L10123^{T} are summarized in Fig. S6A. The genome size of strain EGI L10123^{T} contained 4,107,660 bp with a genomic G+C content of 71.81% and consisted of 1 contig, which harbored 3,984 genes, 3,931 CDSs, 3 rRNAs and 51 tRNAs. Genome-based phylogenetic trees were constructed with methods described before [24]. The phylogenomic tree based on the genomes of strain EGI L10123^{T} and its related pure cultures also supported the phylogenetic relationship based on 16S rRNA genes (Fig. S3A). Amino Acid Identity (AAI) and Average Nucleotide Identity (ANI) values were calculated between genomes of strain EGI L10123^{T} and other species from class *Acidimicrobiia* with FastANI (ver. 1.34) and CompareM (ver. 0.1.2, https://github.com/dparks1134/CompareM), respectively [25]. Strain EGI L10123^{T} had both low ANI and AAI values compared to related type species in this class (Fig. S6B and C).

The genomic properties of strain EGI L10123^{T} and related species are summarized in Table S4. KEGG annotation results showed that strain EGI L10123^{T} had 2,177 genes assigned to 21 KEGG level two pathways. KEGG pathways related to carbohydrate metabolism, amino acid metabolism, and energy metabolism are present in high abundance (Fig. S9A). COG annotation results showed that strain EGI L10123^{T} had 2,838 genes assigned to 22 categories (Fig. S9B). For comparative analyses of the orthologous and exclusive genes between EGI L10123^{T} and the other genomes in the class *Acidimicrobiia*, all protein sequences were filtered to remove low-quality sequences based on length and percent stop codons with the PGCGAP pipeline (ver 1.0.35). Then

these proteomes were compared to each other and to construct gene families using the OrthoFinder program (ver. 2.5.5) [26, 27]. Pan- and core-genome analysis was performed using the Pan program in the PGCGAP pipeline at 40% minimum sequence identity. Comparative genomics analysis based on orthologs showed that strain EGI L10123^T shared only 512 core proteins with other 15 species in this class, whereas 2,083 proteins were exclusive for strain EGI L10123^T (Fig. S10A). At the family level, the results also showed that strain EGI L10123^T shared 1,466, 1,256, 1,404, and 613 core proteins with different families (Fig. S10B-E). The genetic variability among *Acidimicrobiia* species genomes could be further determined from the distribution of the conserved (core) and species-specific (unique) genes. The pan-genome analysis revealed that all genomes comprised 25,857 gene clusters, including 269 homologous gene clusters in the core genome, and that each of the *Acidimicrobiia* species contained a large number of unique genes in their genomes (Fig. S10F). For the cluster of orthologous groups (COGs) analysis, the most abundant COGs in this class were assigned to general function prediction only (COG category R), followed by amino acid transport and metabolism (COG category E) (Fig. S10G). Genomes showed a large difference in the number of genes in each COG category, which suggested that genomes in this class are highly variable.

3. Metabolic traits of strain EGI L10123^T

a) Carbon and energy metabolism

Based on the genome annotation results, strain EGI L10123^T might have carbon fixation ability. Previous studies have shown that *Acidimicrobiia*, as one of the most abundant bacterial groups in both topsoil and biocrusts, was metabolically flexible, and might utilize atmospheric H_2 for aerobic respiration and fix CO_2 via the Calvin-Benson-Bassham (CBB) cycle [28]. However, the carbon fixation pathway of strain EGI L10123^T was different from that described above. The annotation results showed that strain EGI L10123^T might fix CO_2 via the reversed oxidative tricarboxylic acid (roTCA), a highly efficient autotrophic carbon fixation pathway, as key genes (such as *CS*, *gltA*; *por*, *ppdK*, *nifJ*, *korA*, *oorA*, *oforA*; *korB*, *oorB*, *oforB*; and *ppc*) were detected in this metabolic pathway (Fig. 3 and Table S7). Although some genes for other carbon fixation were also annotated from the genome of strain EGI L10123^T, these other pathways were not complete. Hence, this strain might only be able to fix carbon via the reversed oxidative tricarboxylic acid (roTCA). The genome annotation results also revealed a potential microbial rhodopsin gene, and comparative analysis based on the translated protein sequence in the MicRhoDE database showed that this gene had a high score with the actinorhodopsin protein sequence of Actinobacterium_MWH-EgelM2-3_D6 (FJ545222). Subsequently, the phylogenetic relationship of this hypothetical microbial rhodopsin sequence was clarified with related

rhodopsin sequences (Fig. S11). In the phylogenetic tree based on different types of rhodopsin protein sequences, strain EGI L10123^T formed a distinct clade with Superior LG. B2 putative rhodopsin (FJ545146.1) [29], which might be a new type of microbial rhodopsin. In addition, comparison of protein sequences of different microbial rhodopsins showed that the rhodopsin sequence of this isolate and related microbial rhodopsin sequences had some differences in amino acid composition (Fig. S12). These results suggest that this microbial rhodopsin also might utilize light energy to drive a proton motive force to produce ATP and provide extra energy for this strain.

In addition, genes encoding enzymes for carbohydrate metabolism were also found in the genome of strain EGI L10123^T. The analysis showed that strain EGI L10123^T could utilize the Embden-Meyerhof pathway (glycolysis), TCA cycle, and oxidative pentose phosphate pathway to metabolize carbohydrates. At the same time, it could also produce some important metabolites for other metabolic processes, such as the phosphoribosyl diphosphate (PRPP), which in turn could be involved in purine, pyrimidine and histidine metabolism. As a central bridge to other metabolic processes, genes for pyruvate oxidation were also discovered in strain EGI L10123^T. And the metabolite would help this strain to utilize or produce various substrates in the culture niche. For energy metabolism, the genome encodes a complete oxidative phosphorylation pathway, including NADH dehydrogenase (*nuoBDFGHIJKLMN*) for proton translocation, carbon monoxide dehydrogenase (*coxLMS*), cytochrome b6-f complex iron-sulfur subunit (*petC*), cytochrome c oxidase (*ctaCDE*), and F-type ATPase (*atpABCDFGH*). Besides the conventional F-type ATPase, we also identified a copper-exporting P-type ATPase that might function as a Cu^{2+} pump while consuming ATP and a calcium-transporting ATPase that might function as a Ca^{2+} pump while consuming ATP (Fig. S13, Fig. 3, and Table S7).

b) Sulfur, nitrogen, and phosphorus metabolism

This strain also has genes suggesting the ability to utilize other compounds for anabolism or catabolism, including assimilatory sulfate reduction or denitrification. Steps and corresponding genes for assimilatory sulfate reduction include sulfate conversion to adenosine 5'-phosphosulfate (APS) via *cysD*, then to 3'-phosphoadenosine 5'-phosphosulfate (PAPS) via *cysNC*, and finally to sulfite via *cysH*. The resulting sulfite can be converted to sulfide via *cys*I. Then, cysteine synthase (*cysK*) could catalyze hydrogen sulfide and O-acetylserine to synthesize L-cysteine (Fig. S14, Fig. 3, and Table S7). Few genes for nitrogen metabolism were found in the genome of this strain. Nitroalkanes could be degraded to nitrite via nitronate monooxygenase (*npd*), which could be reduced to nitric oxide (NO) by nitrite reductase (*nirK*). Strain EGI L10123^T may be able to transport and assimilate ammonia as a substrate for glutamate synthesis. In addition, strain EGI

L10123^T also encoded a high-affinity ABC transporter for phosphate (*pstABC*), a phosphate-specific transport system accessory protein *phoU*, and an alkaline phosphatase (*phoD*) (Fig. S14, Fig. 3, and Table S7). Furthermore, the API ZYM test results also showed that EGI L10123^T was positive in alkaline phosphatase activity, indicating that this strain might convert phosphorus-containing compounds to inorganic phosphate.

In summary, this strain may participate in carbon, sulfur, nitrogen, and phosphorus cycles for both anabolism and catabolism. To understand the physiology of strain EGI L10123^T, we reconstructed the metabolic pathways and cellular transport systems based on genome annotation (Fig. 3 and Table S7).

4. Morphological, physiological, and chemotaxonomic characterization

Gram staining was tested by using the Gram Stain kit (Solarbio, Beijing) according to the manufacturer's instructions. The motility of strain was tested by inoculating it in a tube filled with semi-solid medium. Strain EGI L10123^T was characterized as Gram-stain positive, non-motile, and facultatively anaerobic. Strain EGI L10123^T can only grow on the marine agar 2216 (Difco^{TM}). Colony characteristics were observed on marine agar 2216 at 37°C after 30 days. The colonies of strain were small, round, neat edge, smooth surface, faint yellow, non-spore-forming. Cell morphology was observed and photographed with a transmission electron microscope (JEM1400FLASH) using the method described previously [30]. Transmission electron micrographs showed that the cells formed long rod-shaped, with 0.4-0.5 µm width and 5.0-10.0 µm length (Fig. 2). Cultural characteristics, temperature, NaCl concentrations and pH range for growth were tested according to a previously published method [31]. All tests were cultured the strain at 37°C for one month. Strain EGI L10123^T grow at 28-37 °C (optimum 37 °C), pH 6.0-10.0 (optimum pH 8.0) and in the presence of 0-5% (w/v) NaCl (optimum 0% (w/v) NaCl).

Catalase activity was detected by production of bubbles with 3% (v/v) H_2O_2 [32]. Oxidase activity was determined using the Oxidase Reagent kit according to the manufacturer's instructions (bioMerieux SA). Milk peptonization and coagulation, urease activity, degradation of cellulose, starch, Tween (20, 40, 60, and 80), and liquefaction of gelatin were performed as described by Smibert and Krieg [33]. Strain EGI L10123^T was negative for catalase, urease, milk peptonization and coagulation, gelatin liquefaction and coagulation, hydrolysis of cellulose, and degradation of Tweens (20, 40, 60, and 80), and starch. However, strain EGI L10123^T was positive for oxidase. Substrate utilization of the target strain was tested at 37 ºC by using GEN III Microplates (BIOLOG Inc., Hayward, CA, United States) according to the manufacturer's instructions. In the Biolog GEN III test results, strain EGI L10123^T utilized D-turanose, *α*-D-lactose, D-galactose, 3-methylglucose, D-fucose, L-fucose, L-rhamnose, D-fructose-6-PO_4, and

glucuronamide. The Biolog GEN III test results also showed that strain EGI L10123^T was positive for vancomycin, α-keto-glutaric acid, and Na-butyrate. Other enzyme activities (including nitrate and nitrite reduction) and biochemical characteristics were tested using API 20NE and API ZYM kits (bioMérieux, France) according to the manufactures' instructions. For enzyme activity of API 20 NE and API ZYM strips, strain EGI L10123^T expressed β-galactosidase, alkaline phosphatase, esterase (C4), esteraselipase (C8), lipase (C14), leucine arylamidase, valine arylamidase, cystine arylamidase, trypsin, α-chymotrypsin, acid phosphatase, Naphthol-AS-BI-phosphohydrolase, and β-glucosidase.

Chemotaxonomic characteristics were also documented. Biomass for the study of chemotaxonomic features was obtained from cultures grown on marine agar 2216 for one month at 37℃. Cellular fatty acid, respiratory quinones, and polar lipids were determined using the methods described in a previous study [30]. The analysis of whole-cell hydrolysis of sugar and the diamino acid of the cell wall was analyzed according to the methods previously described [34]. The major fatty acids (>10%) of strain EGI L10123^T were Summed Feature 8 ($C_{17:1}$ $\omega 6c$ and/or $C_{17:1}$ $\omega 7c$), iso-$C_{16:0}$, anteiso-$C_{14:0}$, and Summed Feature 3 ($C_{16:1}\omega 6c$ and/or $C_{16:1}\omega 7c$) (Table S3). MK-9 (H_8) was found to be the respiratory quinone of strain EGI L10123^T. The polar lipids of strain EGI L10123^T were found to consist of diphosphatidylglycerol (DPG), phosphatidylinositol (PI), phosphatidylinositol mannoside (PIM), phosphatidylethanolamine (PE), phosphatidylglycerol (PG), glycolipid (GL) (Fig. S7). The characteristic diamino acid of strain EGI L10123^T was *meso*-diaminopimelic acid. Additionally, the amino acids alanine, glycine, and glutamic acid were detected in hydrolysates of the cell wall peptidoglycan. Sugars found in whole cell hydrolysates were galactose, mannose, and rhamnose.

All detailed phenotypic, physiological and biochemical characteristics that differentiate strain EGI L10123^T from the reference type strain of the class *Acidimicrobiia* are listed in supplementary excel file (Table S3). We propose this isolate, strain EGI L10123^T as a new member of new family *Salinilacustritrichaceae* fam. nov, in the class *Acidimicrobiia* for which the name *Salinilacustristhrix flava* sp. nov. is proposed with EGI L10123^T (=CGMCC 1.19137^T= KCTC 49680^T) as the type strain.

Supplementary Figures

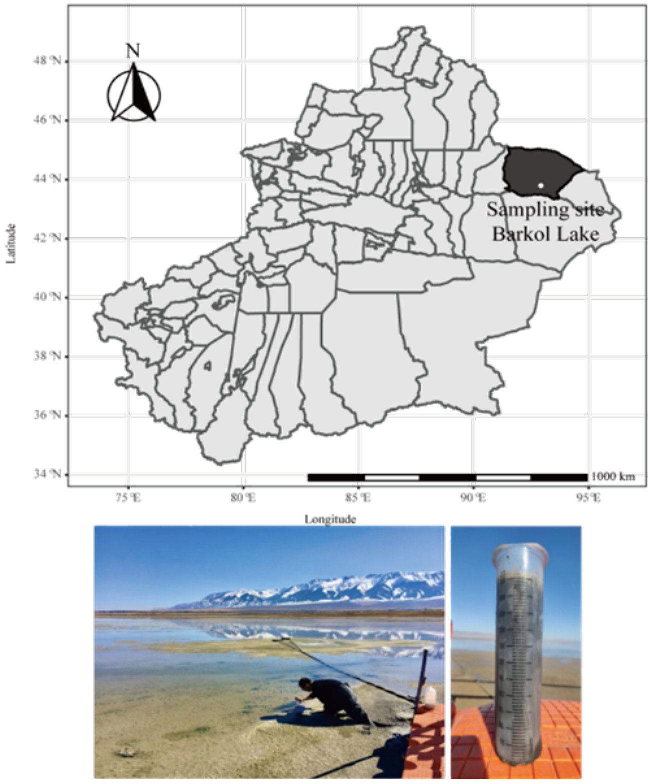

Fig. S1 Sampling site and sample collection.

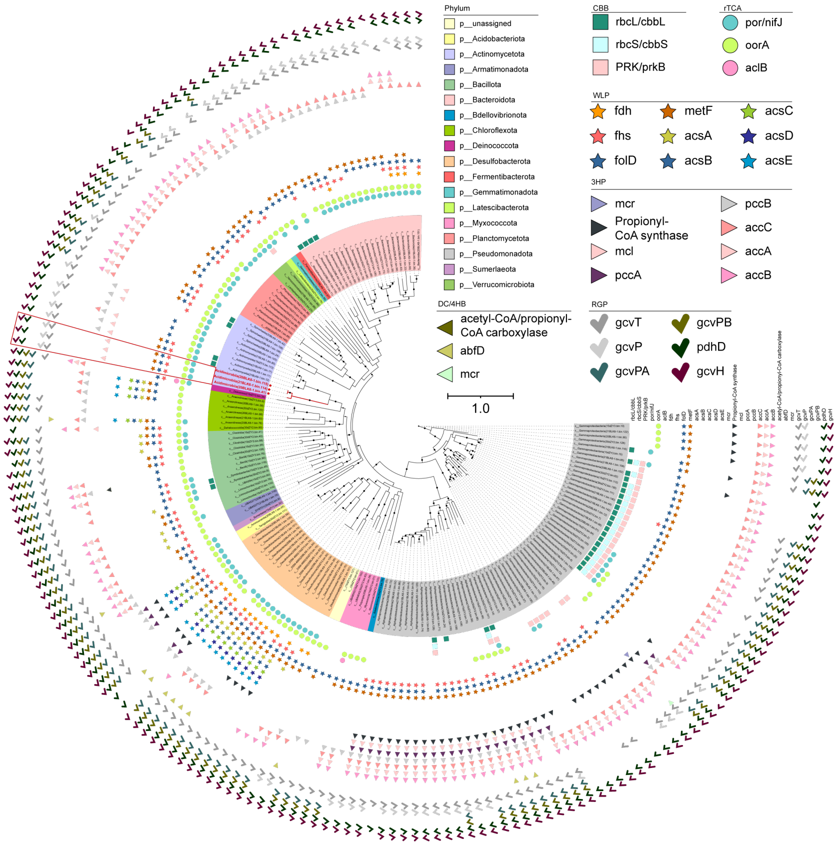

Fig. S2 Key genes of carbon fixation pathways distribution in 171 high-quality MAGs. The phylogenetic relationships were inferred using 120 bacteria marker genes by GTDB-Tk. Bootstrap values (> 90%) based on 1000 resamplings are given at the nodes. Color ranges identify phyla within the tree. Colored symbols indicate different carbon fixation pathways.

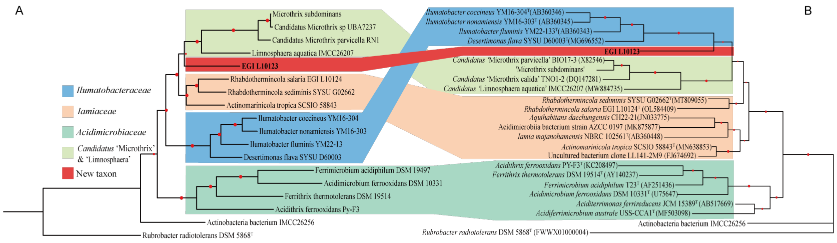

Fig. S3 Phylogenetic analysis of strain EGI L10123^{T} with type species in the order *Acidimicrobiales*. A: Phylogenomic tree of strain EGI L10123^{T} and related type species of the order *Acidimicrobiales*. Bootstrap values (> 50%) based on 1000 resampling are given at the nodes. *Rubrobacter radiotolerans* DSM 5868^{T} was used as the outgroup. The phylogenetic relationships were inferred using 120 bacteria marker genes identified by GTDB-Tk. B: Maximum-likelihood phylogenetic tree based on 16S rRNA gene sequences showing the relationship between strain EGI L10123^{T} and related type species. Bootstrap values (> 50%) based on 1000 resamplings are given at the nodes. *Rubrobacter radiotolerans* DSM 5868^{T} (FWWX01000004) was used as the outgroup.

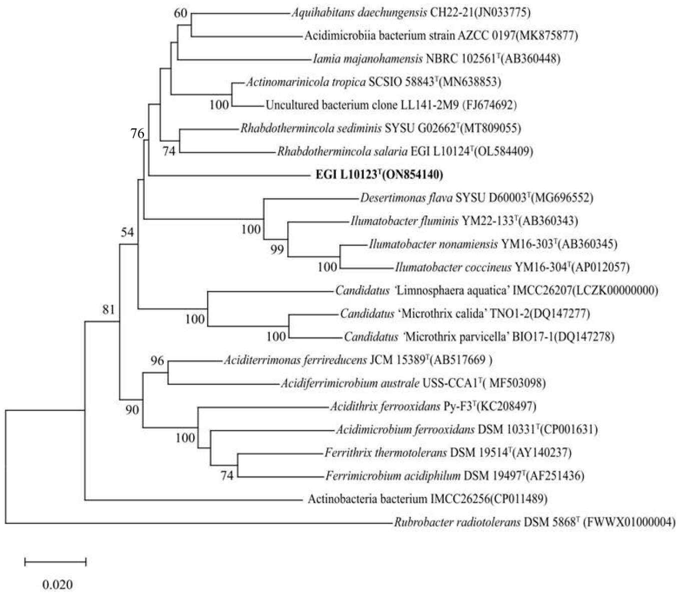

Fig. S4 Neighbor-joining (NJ) phylogenetic tree based on 16S rRNA gene sequences showing the relationship of strain EGI L10123^{T} with other type species in the order *Acidimicrobiales*. The phylogenetic tree was constructed using MEGA X based on the Kimura 2-parameter model. *Rubrobacter radiotolerans* DSM 5868^{T} (FWWX01000004) was used as the outgroup. Bootstrap values (based on the percentages of 1,000 replications) over 50% are shown at branch nodes. Bar, 0.02 substitutions per nucleotide position.

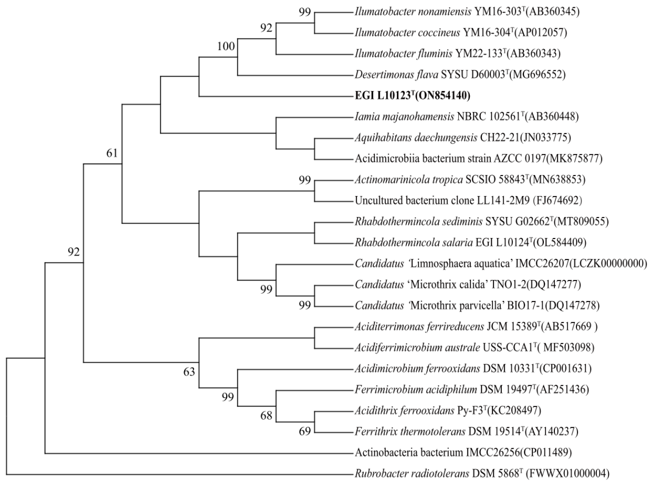

Fig. S5 Maximum-Parsimony phylogenetic tree based on 16S rRNA gene sequences showing the position of strain EGI L10123^T among the members of the class *Acidimicrobiia*. Bootstrap values of above 50% are shown at the branch points. *Rubrobacter radiotolerans* DSM 5868^T (FWWX01000004) was used as the outgroup.

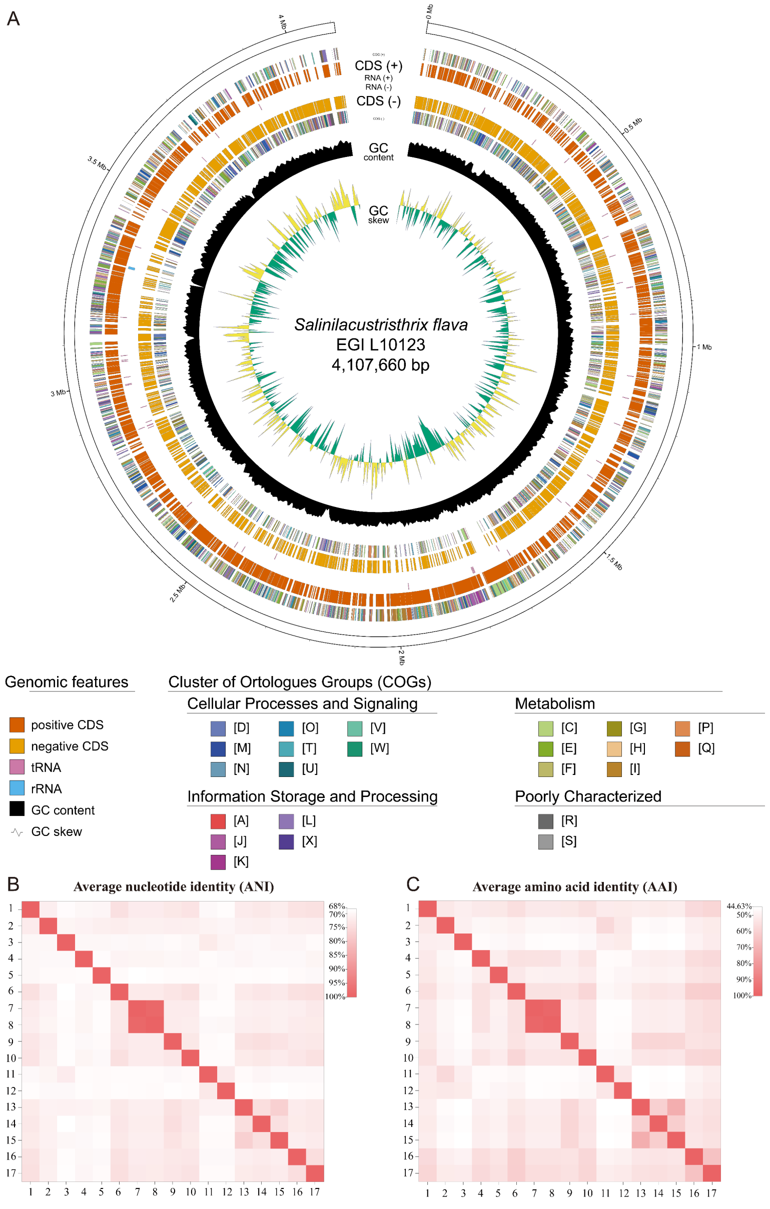
A
CDS (+)
RNA (+)
RNA (-)
CDS (-)
GC content
GC skew
0 Mb
0.5 Mb
1 Mb
1.5 Mb
2 Mb
2.5 Mb
3 Mb
3.5 Mb
4 Mb
Salinilacustristhrix flava
EGI L10123
4,107,660 bp
Genomic features
positive CDS
negative CDS
tRNA
rRNA
GC content
GC skew
Cluster of Ortologues Groups (COGs)
Cellular Processes and Signaling
[D] [O] [V]
[M] [T] [W]
[N] [U]
Metabolism
[C] [G] [P]
[E] [H] [Q]
[F] [I]
Information Storage and Processing
[A] [L]
[J] [X]
[K]
Poorly Characterized
[R]
[S]
B
Average nucleotide identity (ANI)
68%
70%
75%
80%
85%
90%
95%
100%
1 2 3 4 5 6 7 8 9 10 11 12 13 14 15 16 17
C
Average amino acid identity (AAI)
44.63%
50%
60%
70%
80%
90%
100%
1 2 3 4 5 6 7 8 9 10 11 12 13 14 15 16 17

Fig. S6 A, Genomic architecture of EGI L10123^{T}. Each ring, from outside to the center represented the following features: scale marks of the genome, protein-coding genes on the forward strand, protein-coding genes on the reverse strand, tRNA (black) and rRNA (pink) genes on the forward strand, tRNA (black) and rRNA (red) genes on the reverse strand, GC content, and GC content skew. B, Average nucleotide identity (ANI) values shared among the related type species. C, Average amino acid identity (AAI) values shared among the related type species. Notes: 1, *Acidiferrimicrobium australe* USS-CCA1^{T}; 2, *Acidimicrobium ferrooxidans* DSM 10331^{T}; 3, *Acidithrix ferrooxidans* Py-F3^{T}; 4, Actinobacteria bacterium IMCC26207; 5, Actinobacteria bacterium IMCC26256; 6, *Actinomarinicola tropica* SCSIO 58843^{T}; 7, *Candidatus* ‘Microthrix parvicella’ Bio17-1; 8, *Candidatus* ‘Microthrix parvicella’ RN1; 9, *Desertimonas flava* SYSU D60003^{T}; **10, EGI L10123^{T}**; 11, *Ferrimicrobium acidiphilum* DSM 19497^{T}; 12, *Ferrithrix thermotolerans* DSM 19514^{T}; 13, *Ilumatobacter coccineus* YM16-304^{T}; 14, *Ilumatobacter fluminis* DSM 18936^{T}; 15, *Ilumatobacter nonamiensis* YM16-303^{T}; 16, *Rhabdothermincola sediminis* SYSU G02662^{T}; 17, *Rhabdothermincola salaria* EGI L10124^{T}.

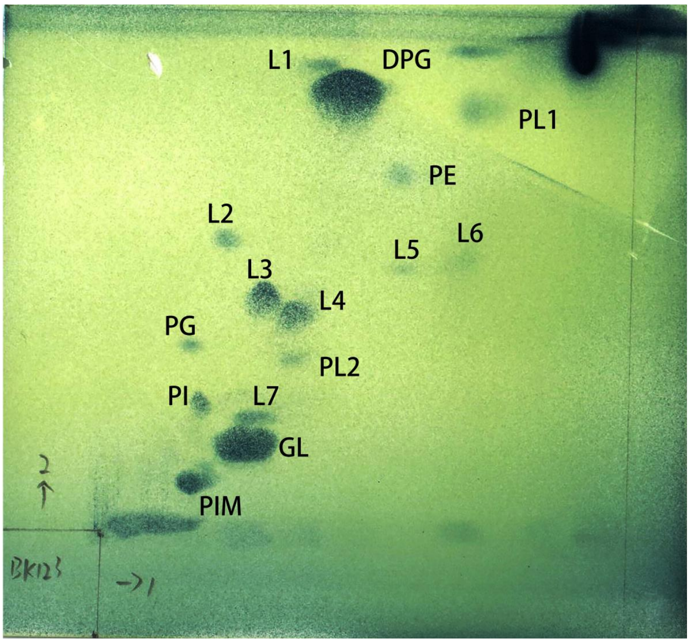

Fig. S7 Two-dimensional thin-layer chromatogram of polar lipids of strain EGI L10123^T following staining with 5% ethanolic molybdophosphoric acid. The chromatographic conditions were as follows: Silica Gel 60 thin-layer plates (10 by 10 cm) were spotted with 15 µL of a whole-cell lipid extract. Chloroform-methanol-water (65:25:4, vol/vol/vol) was used to develop the chromatogram in the first direction, and chloroform-acetic acid-methanol-water (80:18:12:5, vol/vol/vol/vol) was used in the second direction.

Abbreviations: DPG, diphosphatidylglycerol; PI, phosphatidylinositol; PIM, phosphatidylinositol mannoside; PE, phosphatidylethanolamine; PG, phosphatidylglycerol; GL, glycolipid; PL, unidenified phospholipid; and L, unidenified lipid.

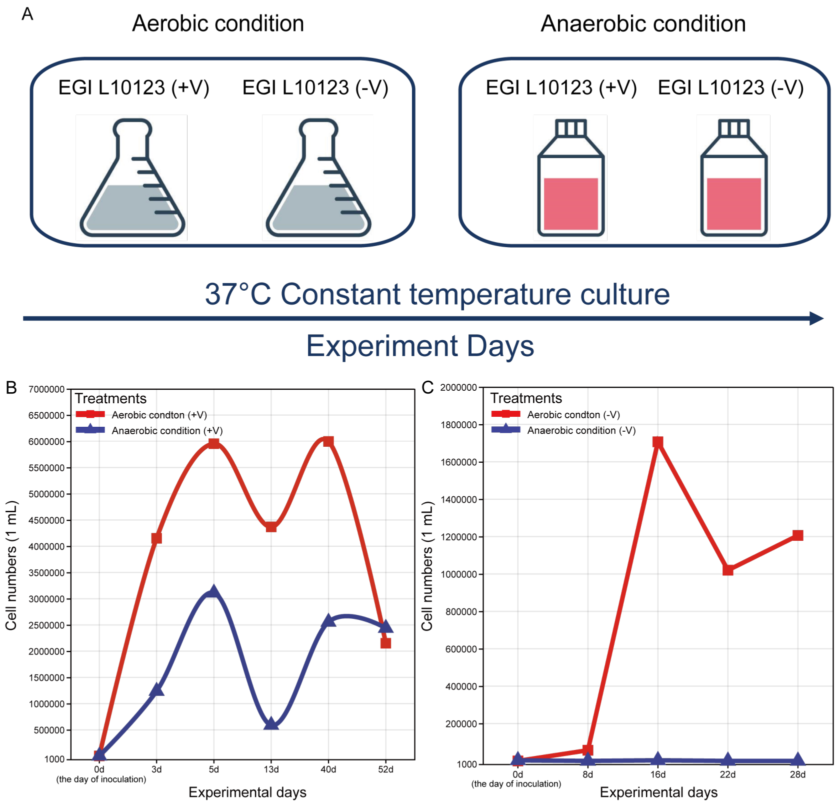

Fig. S8 Chemoautotrophic function verification of strain EGI L10123^T via flow cytometry under different conditions.

Notes: +V, the verification experiment in the M3 medium added with vitamin solution; -V, the verification experiment in the M3 medium without adding vitamin solution.

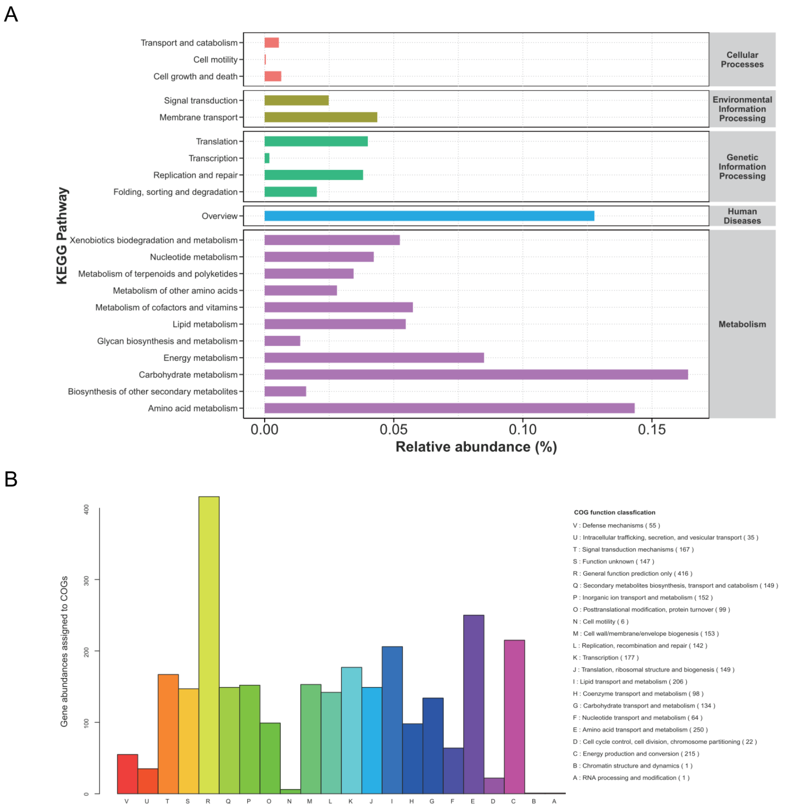

Fig. S9 KEGG pathway abundance (A) and COG functional classifications (B) of strain EGI L10123^{T}.

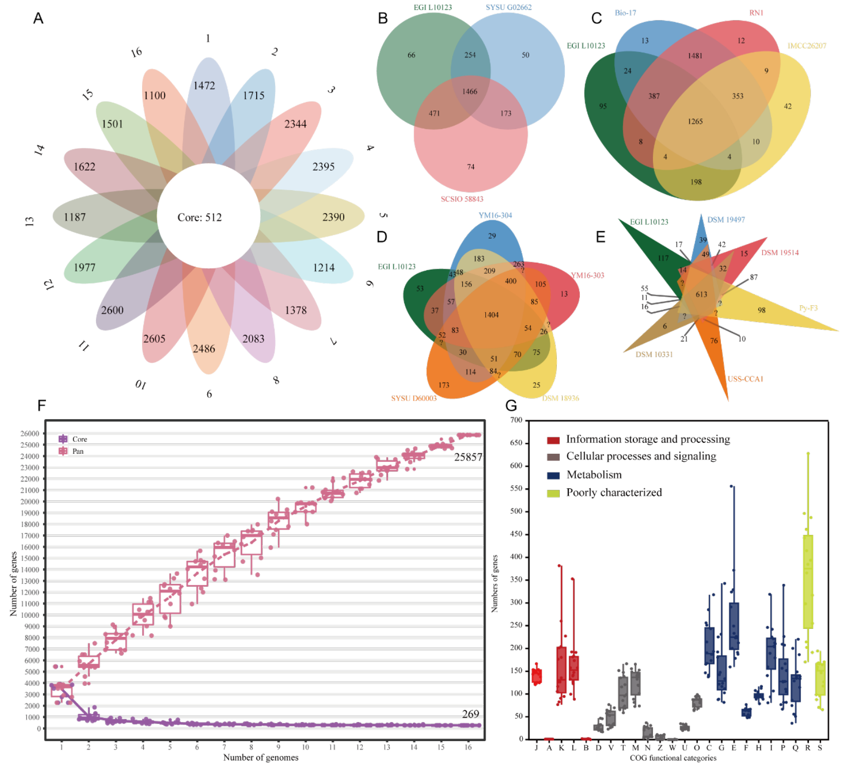

Fig. S10 Comparison of the genome of strain EGI L10123^{T} with the genomes of the class *Acidimicrobiia*. The flower and Venn diagrams (A-E) illustrate the number of unique and shared protein-coding genes among all *Acidimicrobiia* strain genomes. F, Pan- and core-genome evolution of *Acidimicrobiia*. G, Categorization of the function of each protein-coding gene was based on COG categories.

Note: 1, *Acidiferrimicrobium australe* USS-CCA1^{T}; 2, *Rhabdothermincola sediminis* SYSU G02662^{T}; 3, *Ilumatobacter nonamiensis* YM16-303^{T}; 4, *Ilumatobacter fluminis* DSM 18936^{T}; 5, *Ilumatobacter coccineus* YM16-304^{T}; 6, *Ferrithrix thermotolerans* DSM 19514^{T}; 7, *Ferrimicrobium acidiphilum* DSM 19497^{T}; **8, EGI L10123^{T}**; 9, *Desertimonas flava* SYSU D60003^{T}; 10, *Candidatus* 'Microthrix parvicella' RN1; 11, *Candidatus* 'Microthrix parvicella' Bio17-1; 12, *Actinomarinicola tropica* SCSIO 58843^{T}; 13, Actinobacteria bacterium IMCC26256; 14, Actinobacteria bacterium IMCC26207; 15, *Acidithrix ferrooxidans* Py-F3^{T}; 16, *Acidimicrobium ferrooxidans* DSM 10331^{T}.

A, RNA processing and modification; B, Chromatin structure and dynamics; C, Energy production and conversion; D, Cell cycle control, cell division, chromosome partitioning; E, Amino acid transport and metabolism; F, Nucleotide transport and metabolism; G, Carbohydrate transport and metabolism; H, Coenzyme transport and metabolism; I, Lipid transport and metabolism; J, Translation, ribosomal structure and biogenesis; K, Transcription; L, Replication, recombination and repair; M, Cell wall/membrane/envelope biogenesis; N, Cell motility; O, Posttranslational modification, protein turnover; P, Inorganic ion transport and metabolism; Q, Secondary metabolites biosynthesis, transport and catabolism; R, General function prediction only; S, Function unknown; T, Signal transduction mechanisms; U, Intracellular trafficking, secretion, and vesicular transport; V, Defense mechanisms; W, Extracellular structures; Z, Cytoskeleton.

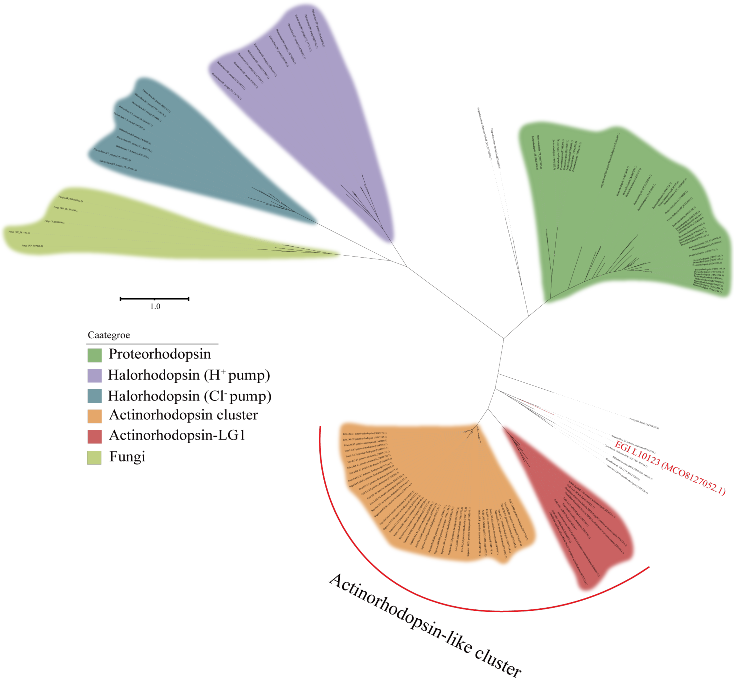

Fig. S11 Phylogenetic relationship of the hypothetical microbial rhodopsin of EGI L10123^T and related microbial rhodopsins. Best maximum-likelihood tree of 129 selected microbial rhodopsin proteins. Only selected bootstrap values are displayed. Major clades of rhodopsin sequences are outlined.

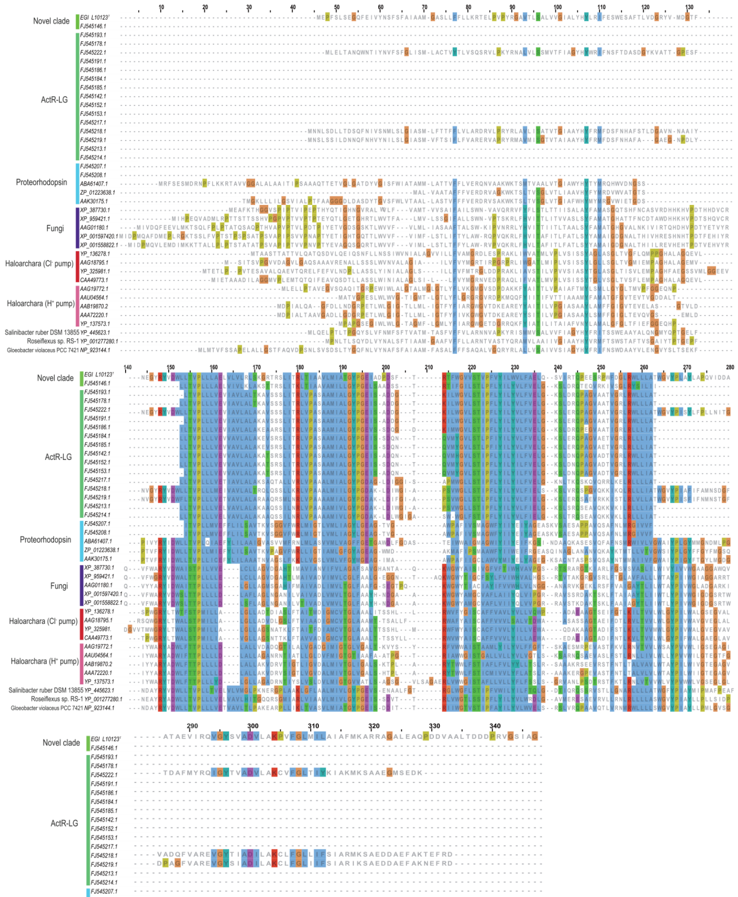

Fig. S12 Comparison of the hypothetical microbial rhodopsin of EGI L10123^T and related microbial rhodopsins.

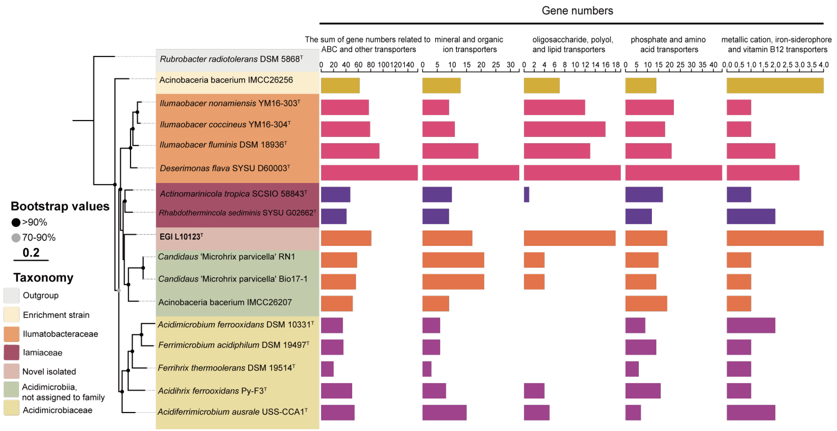

Fig. S13 Energy metabolism of strain EGI L10123^{T} and closely related strains of the class *Acidimicrobiia*. Phylogenomic tree of strain EGI L10123^{T} and related genomes of the class *Acidimicrobiia.* Bootstrap values (> 70%) based on 1000 resamplings are marked at the nodes. Colored bars represent the numbers of genes related to transporters of each strain.

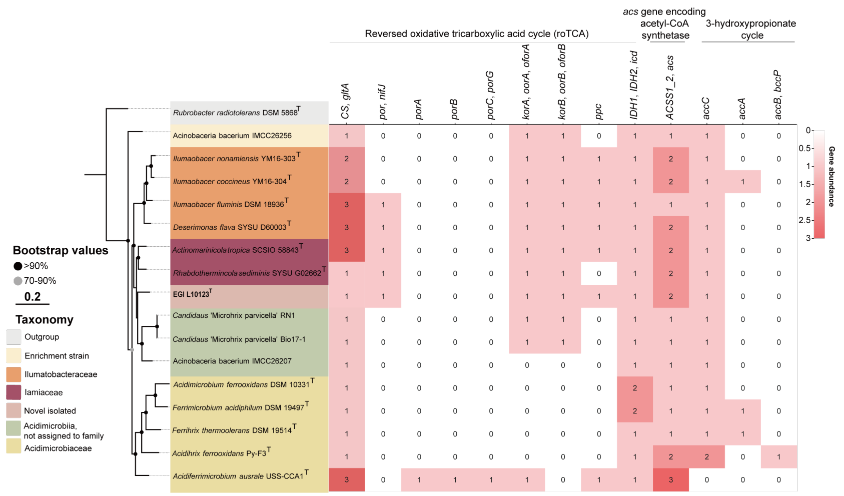

Fig. S14 Some nitrogen and sulfur metabolism genes in strain EGI L10123^T and closely related strains of the class *Acidimicrobiia*. Phylogenomic tree of strain EGI L10123^T and related genomes of the class *Acidimicrobiia.* Bootstrap values (> 70%) based on 1000 resamplings are marked at the nodes. Color ranges identify taxonomy within the tree. Heatmap represents the gene numbers related to nitrite reductase and assimilatory sulfate reduction of each strain.

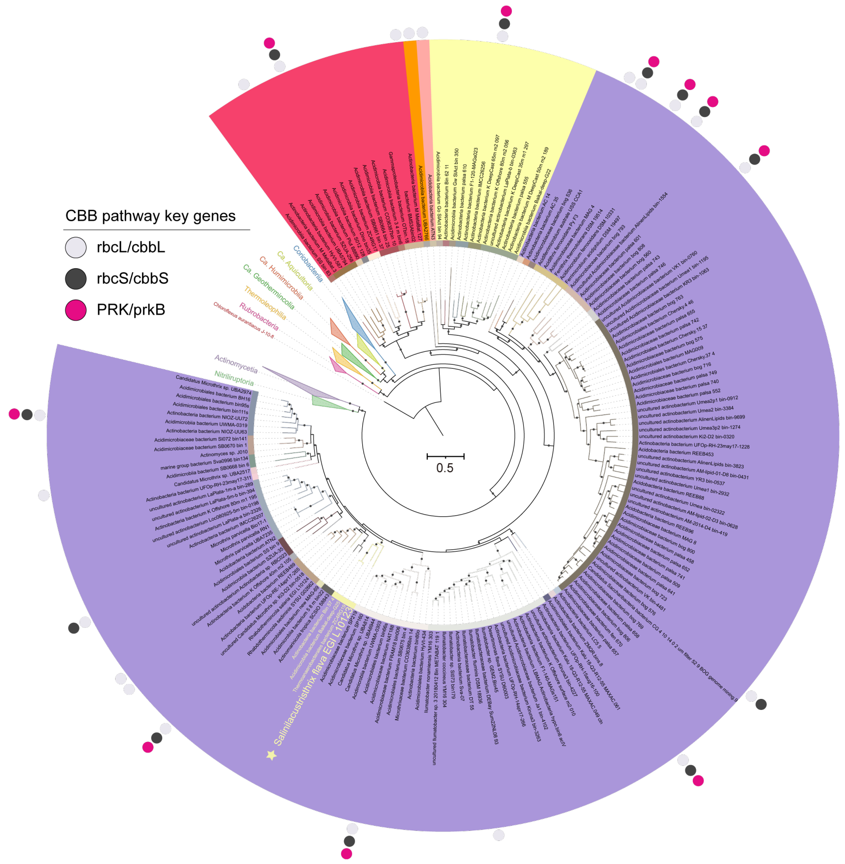

Fig. S15 Distribution patterns of key genes associated with the CBB cycle within *Acidimicrobiia*. The scale bar represents the number of substitutions per site. Closed black circles indicate bootstrap support values > 90%.

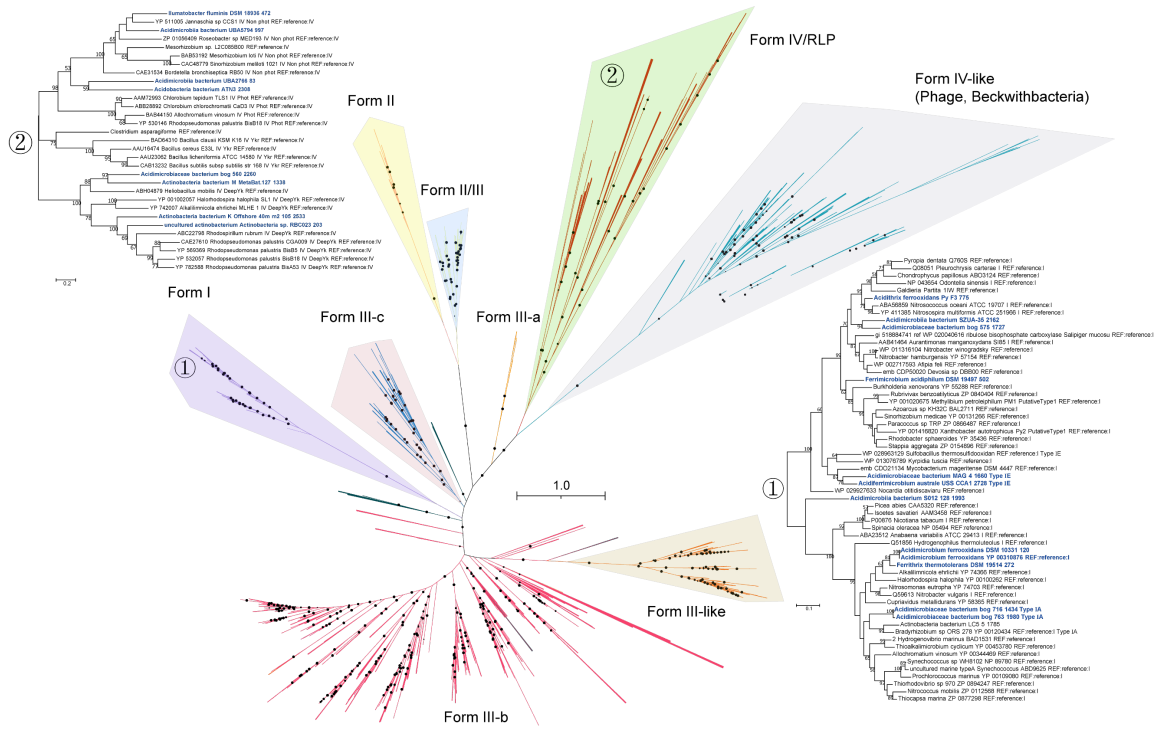

Form IV/RLP
Form IV-like
(Phage, Beckwithbacteria)
Form II
Form II/III
Form I
Form III-c
Form III-a
1.0
Form III-like
Form III-b

Fig. S16 Maximum-likelihood tree for dereplicated RuBisCO large-chain sequences (*rbcL*), delineated by “Form”. The scale bar represents the number of substitutions per site. Closed black circles indicate bootstrap support values > 90%; Colored font indicates the *rbcL* gene of *Acidimicrobiia*.

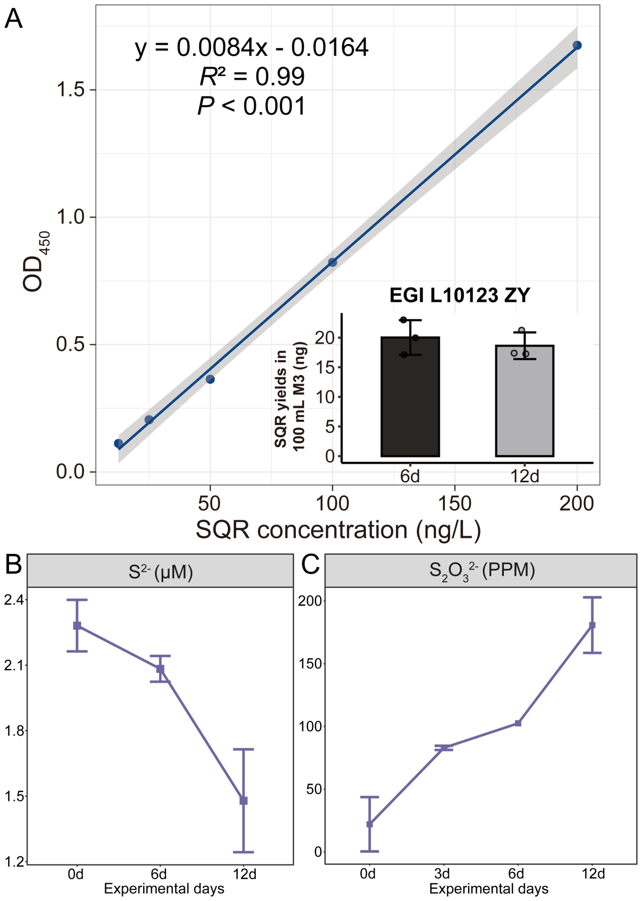

Fig. S17 A, Sulfide: quinone oxidoreductase (SQR) production of strain EGI L10123^T in 100 mL chemolithoautotrophic (M3) medium. B, The variation of S^{2-} concentration in the chemolithoautotrophic system at different time points. C, The variation of $S_2O_3^{2-}$concentration in the chemolithoautotrophic system at different time points. Linear regression is a statistical approach for constructing the standard curve used to quantify SQR concentrations within samples. The bar plot illustrates the SQR yields of strain EGI L10123^T in 100 mL of M3 medium after 6 days and 12 days incubation under 37°C.

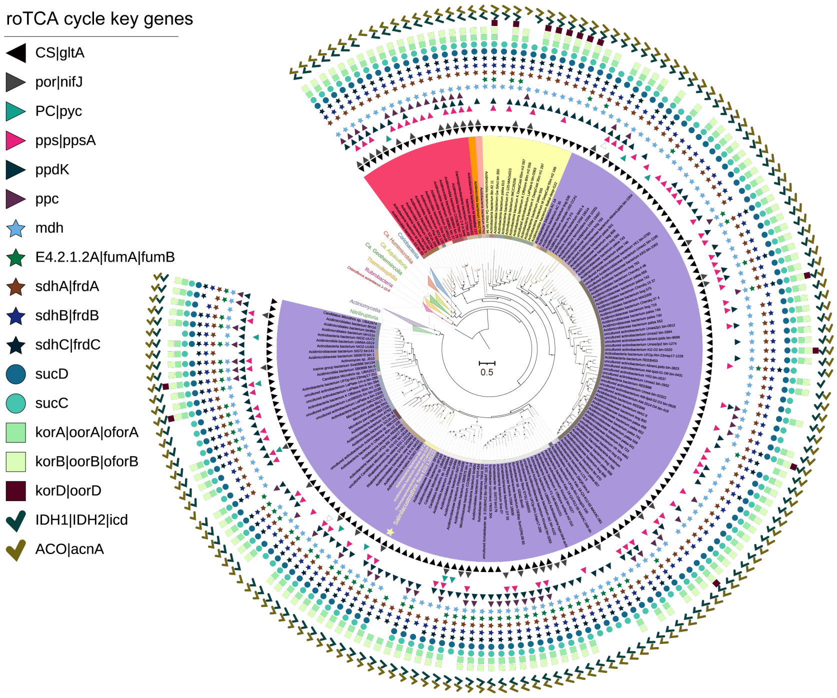

Fig. S18 Distribution patterns of key genes associated with the roTCA within *Acidimicrobiia*. The scale bar represents the number of substitutions per site. Closed black circles indicate bootstrap support values > 90%. Strain EGI L10123^{T} is shown with a yellow star. Different shapes indicate different genes in the roTCA.

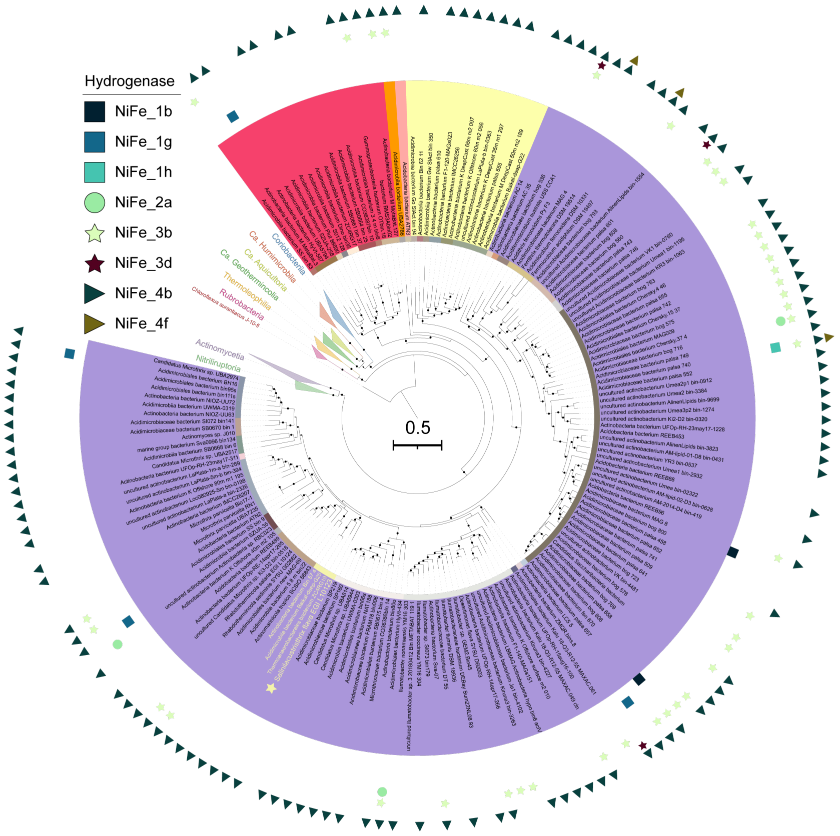

Fig. S19 Distribution patterns of hydrogenase genes associated with the class *Acidimicrobiia*. The scale bar represents the number of substitutions per site. The black circles indicate bootstrap support values > 95%. Different shapes and colors show the different type of hydrogenases. Strain EGI L10123^{T} is shown with a yellow star. Different shapes indicate different genes in the roTCA.

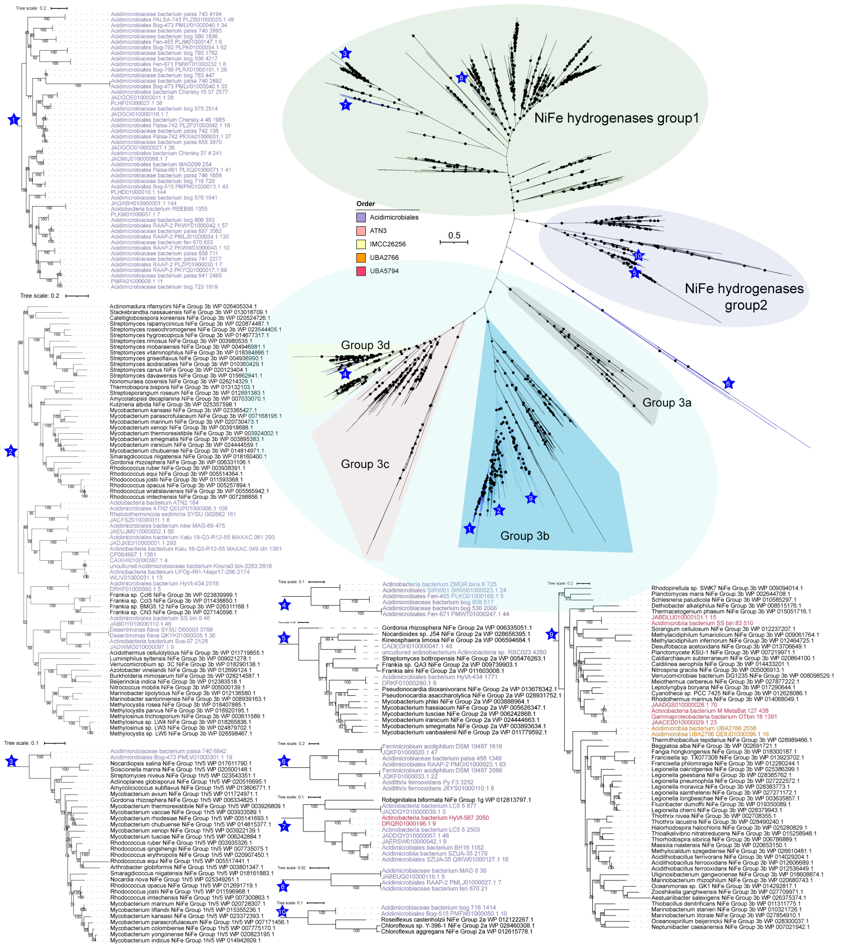

Fig. S20 Phylogenetic tree of groups 1, 2 and 3 [NiFe] hydrogenases catalytic subunits. Hydrogenase genes of the *Acidimicrobiia* recovered in this study are shown with a blue star. Different font colors represent different orders in class *Acidimicrobiia*. Bootstrap values > 90% were shown on nodes. Scale bar indicates substitutions per site.

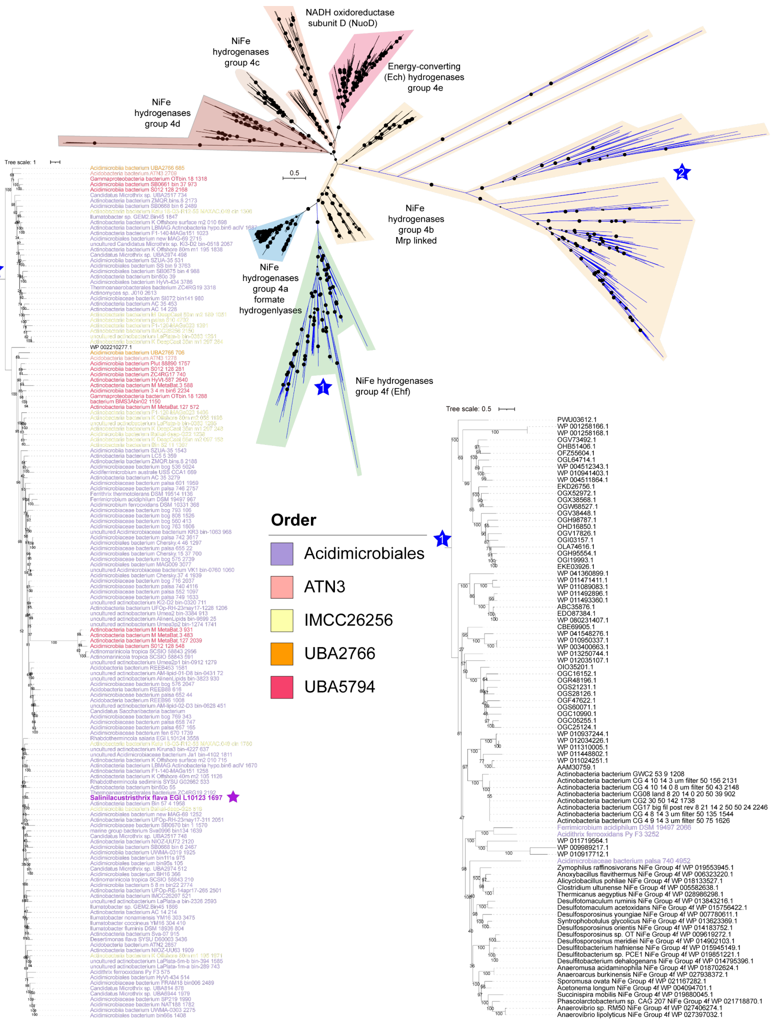

Fig. S21 Phylogenetic tree of group 4 [NiFe] hydrogenases catalytic subunits. Hydrogenase genes of the *Acidimicrobiia* recovered in this study are shown with a blue star. Different font colors represent different orders in class *Acidimicrobiia*. Bootstrap values > 90% were shown on nodes. Scale bar indicates substitutions per site. [NiFe] hydrogenases group 4 of strain EGI L10123^{T} are shown with a deep blue star.

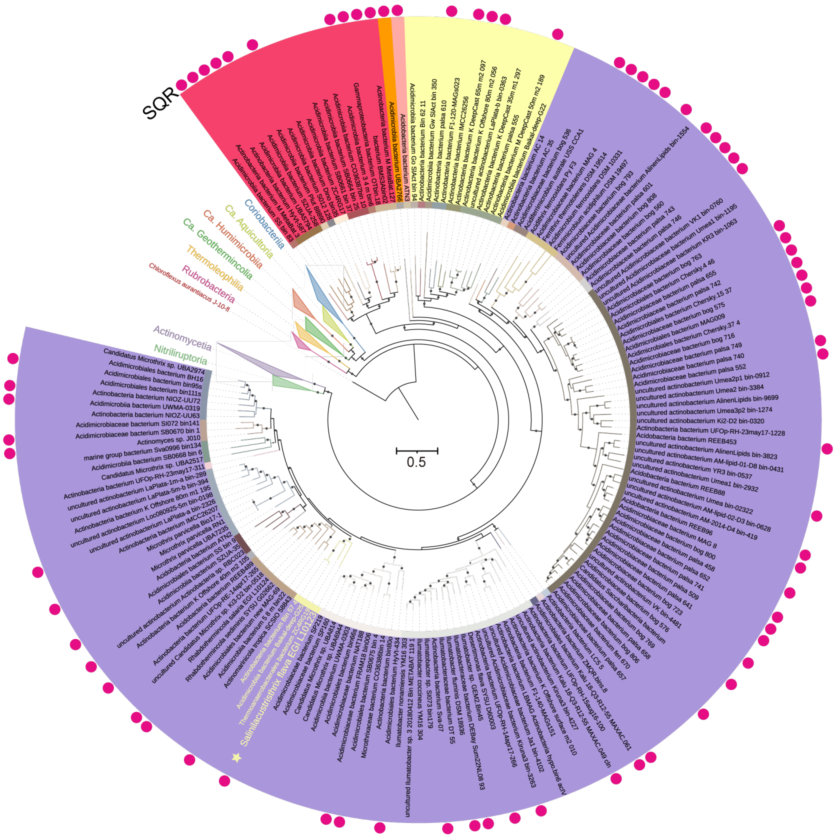

Fig. S22 Distribution patterns of *SQR* genes within *Acidimicrobiia*. The scale bar represents the number of substitutions per site. Closed black circles indicate bootstrap support values > 90%. Strain EGI L10123^{T} is shown with a yellow star.

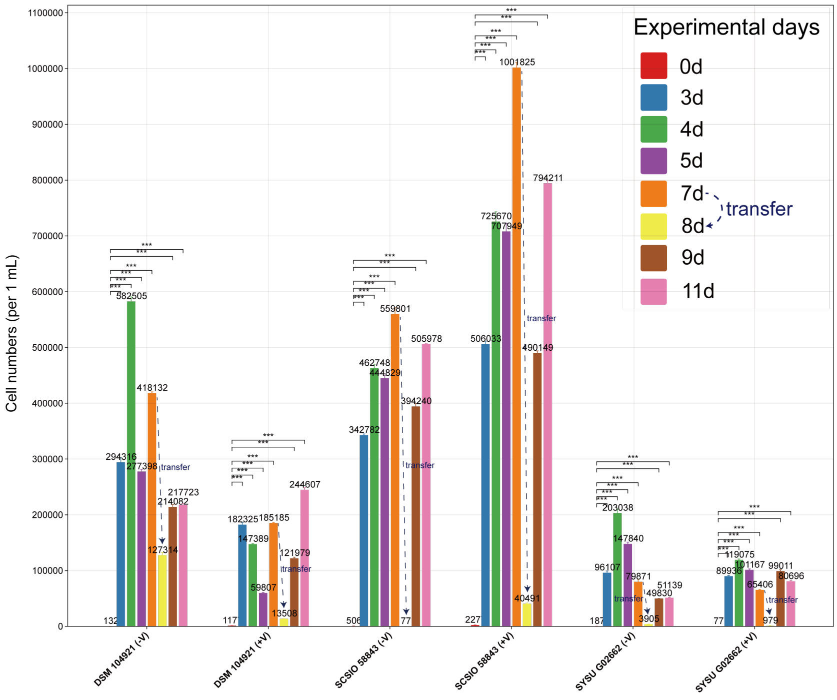

Fig. S23 Results of the chemolithoautotrophic growth experiments for three additional *Acidimicrobia* strains.

Notes: These strains were selected due to their lack of the Calvin-Benson-Bassham (CBB) cycle and other known carbon fixation pathways, while possessing all the necessary genes associated with the roTCA. DSM 149021^{T}: *Desertimonas flava*, SCSIO 58843^{T}: *Actinomarinicola tropica*, SYSU G02662^{T}: *Rhabdothermincola sediminis*, ***: Indicates significant difference ($P < 0.001$), +V: M3 medium added with vitamin solution, -V: M3 medium without adding vitamin solution, transfer: culture transferred to fresh chemolithoautotrophic medium.

Reference

1. Assié A, Leisch N, Meier DV, Gruber-Vodicka H, Tegetmeyer HE, et al. Horizontal acquisition of a patchwork calvin cycle by symbiotic and free-living *Campylobacterota* (formerly *Epsilonproteobacteria*). ISME J. 2020;14:104-122.
2. Garritano AN, Song W, Thomas T. Carbon fixation pathways across the bacterial and archaeal tree of life. PNAS Nexus. 2022;1:pgac226.
3. Srinivasan V, Morowitz HJ. Ancient genes in contemporary persistent microbial pathogens. Biol Bull. 2006;210:1-9.
4. Murphy CL, Biggerstaff J, Eichhorn A, Ewing E, Shahan R, et al. Genomic characterization of three novel *Desulfobacterota* classes expand the metabolic and phylogenetic diversity of the phylum. Environ Microbiol. 2021;23:4326-4343.
5. Fang BZ, Han MX, Jiao JY, Xie YG, Zhang XT, et al. *Streptomyces cavernae* sp. nov., a novel actinobacterium isolated from a karst cave sediment sample. Int J Syst Evol Microbiol. 2020;70:120-125.
6. Yoon SH, Ha SM, Kwon S, Lim J, Kim Y, et al. Introducing EzBioCloud: a taxonomically united database of 16S rRNA gene sequences and whole-genome assemblies. Int J Syst Evol Microbiol. 2017;67:1613-1617.
7. Altschul SF, Gish W, Miller W, Myers EW, Lipman DJ. Basic local alignment search tool. J Mol Biol. 1990;215:403-410.
8. Konstantinidis KT, Rosselló-Móra R, Amann R. Uncultivated microbes in need of their own taxonomy. ISME J. 2017;11:2399-2406.
9. Thompson JD, Gibson TJ, Plewniak F, Jeanmougin F, Higgins DG. The CLUSTAL_X windows interface: flexible strategies for multiple sequence alignment aided by quality analysis tools. Nucleic Acids Res. 1997;25:4876-4882.
10. Kumar S, Stecher G, Tamura K. MEGA7: molecular evolutionary genetics analysis version 7.0 for bigger datasets. Mol Biol Evol. 2016;33:1870-1874.
11. Casanellas M, Fernández-Sánchez J, Roca-Lacostena J. Embeddability and rate identifiability of Kimura 2-parameter matrices. J Math Biol. 2020;80:995-1019.
12. Saitou N, Nei M. The neighbor-joining method: a new method for reconstructing phylogenetic trees. Mol Biol Evol. 1987;4:406-425.
13. Prins N. Too much model, too little data: How a maximum-likelihood fit of a psychometric function may fail, and how to detect and avoid this. Atten Percept Psychophys. 2019;81:1725-1739.
14. Mawhorter R, Libeskind-Hadas R. Hierarchical clustering of maximum parsimony

reconciliations. BMC Bioinformatics. 2019;20:612.
15. Wick RR, Judd LM, Gorrie CL, Holt KE. Unicycler: Resolving bacterial genome assemblies from short and long sequencing reads. PLoS Comput Biol. 2017;13:e1005595.
16. Hyatt D, Chen GL, Locascio PF, Land ML, Larimer FW, et al. Prodigal: prokaryotic gene recognition and translation initiation site identification. BMC Bioinformatics. 2010;11:119.
17. Chan PP, Lowe TM. tRNAscan-SE: Searching for tRNA Genes in Genomic Sequences. Methods Mol Biol. 2019;1962:1-14.
18. Lagesen K, Hallin P, Rødland EA, Staerfeldt HH, Rognes T, et al. RNAmmer: consistent and rapid annotation of ribosomal RNA genes. Nucleic Acids Res. 2007;35:3100-3108.
19. Galperin MY, Wolf YI, Makarova KS, Vera Alvarez R, Landsman D, et al. COG database update: focus on microbial diversity, model organisms, and widespread pathogens. Nucleic Acids Res. 2021;49:D274-d281.
20. Kanehisa M, Goto S. KEGG: kyoto encyclopedia of genes and genomes. Nucleic Acids Res. 2000;28:27-30.
21. Buchfink B, Xie C, Huson DH. Fast and sensitive protein alignment using DIAMOND. Nat Methods. 2015;12:59-60.
22. Möller S, Croning MD, Apweiler R. Evaluation of methods for the prediction of membrane spanning regions. Bioinformatics. 2001;17:646-653.
23. Cumsille A, Durán RE, Rodríguez-Delherbe A, Saona-Urmeneta V, Cámara B, et al. GenoVi, an open-source automated circular genome visualizer for bacteria and archaea. PLoS Comput Biol. 2023;19:e1010998.
24. Jiao JY, Fu L, Hua ZS, Liu L, Salam N, et al. Insight into the function and evolution of the Wood–Ljungdahl pathway in *Actinobacteria*. ISME J. 2021;15:3005-3018.
25. Jain C, Rodriguez RL, Phillippy AM, Konstantinidis KT, Aluru S. High throughput ANI analysis of 90K prokaryotic genomes reveals clear species boundaries. Nat Commun. 2018;9:5114.
26. Emms DM, Kelly S. OrthoFinder: phylogenetic orthology inference for comparative genomics. Genome Biol. 2019;20:238.
27. Liu H, Xin B, Zheng J, Zhong H, Sun M. Build a Bioinformatic Analysis Platform and Apply it to Routine Analysis of Microbial Genomics and Comparative Genomics. 2021.
28. Bay SK, Waite DW, Dong X, Gillor O, Chown SL, et al. Chemosynthetic and photosynthetic bacteria contribute differentially to primary production across a steep desert aridity gradient. ISME J. 2021;15:3339-3356.

29. Sharma AK, Sommerfeld K, Bullerjahn GS, Matteson AR, Wilhelm SW, et al. Actinorhodopsin genes discovered in diverse freshwater habitats and among cultivated freshwater *Actinobacteria*. ISME J. 2009;3:726-737.
30. Fang BZ, Gao L, Jiao JY, Zhang ZT, Li MM, et al. *Agromyces cavernae* sp. nov., a novel member of the genus *Agromyces* isolated from a karstic cave in Shaoguan. Int J Syst Evol Microbiol. 2022;72.
31. Gao L, Fang BZ, Liu YH, Jiao JY, Li MM, et al. *Rhabdothermincola salaria* sp. nov., a novel actinobacterium isolated from a saline lake sediment. Int J Syst Evol Microbiol. 2022;72.
32. Williams S. Genus *Streptomyces* Waksman and Henrici 1943. Bergeys Manual of Systematic Bacteriology. 1989;4.
33. Smibert RM, Krieg NR. Phenotypic characterization.In methods for general and molecular bacteriology. 1994.
34. Hasegawa T, Takaizawa M, Tanida S. A rapid analysis for chemical grouping of aerobic actinomycetes. J. Gen. Appl. Microbiol. 1983;29:319-322.
